# Development and preliminary experimental evaluation of a venturi-based slurry jet erosion test rig for comparative study of ductile and brittle materials

**DOI:** 10.1371/journal.pone.0355225

**Published:** 2026-07-31

**Authors:** Karthik S, Sharath B N, Abhishek Agarwal, Madhu P, Yashas Gowda T G, Rajesh T N

**Affiliations:** 1 Department of Mechanical Engineering, Aditya College of Engineering and Technologyhra Pradesh, Karnataka, India; 2 Department of Mechanical Engineering, Malnad College of Engineering, Hassan, Karnataka, India; 3 Department of Mechanical Engineering, College of Science and Technology, Royal University of Bhutan, Thimphu, Bhutan; 4 Department of Mechanical Engineering, Coorg Institute of Technology, Ponnampet, Affiliated to Visvesvaraya Technological University, Belagavi, Karnataka, India; Ramaiah Institute of Technology, INDIA

## Abstract

Slurry erosion significantly affects the durability of fluid-handling components in hydroelectric, mining, petroleum, and chemical industries. This study presents the design and preliminary laboratory-scale evaluation of a Venturi-based slurry jet erosion test rig capable of introducing dry erosive particles into a flowing liquid stream without the use of pressurised or vacuum systems. The erosion behaviour of brass (ductile) and cast iron (brittle) was investigated under varying particle sizes, slurry concentrations, and impingement angles using river sand as the erodent. Results showed maximum erosion of brass at 30° due to dominant plastic deformation, whereas cast iron exhibited peak erosion at 90° due to brittle fracture mechanisms. Kinetic energy threshold values required for erosion initiation were estimated for both materials and correlated with experimental observations. Qualitative surface morphology analysis using scanning electron microscopy supported the identification of distinct erosion mechanisms governing the ductile and brittle material responses. The developed apparatus demonstrated reasonably consistent operation and repeatable comparative erosion behaviour under the selected experimental conditions, indicating its suitability for controlled slurry erosion testing and comparative evaluation of ductile and brittle engineering materials without the need for pressurised particle delivery systems.

## 1 Introduction

Material deterioration from slurry erosion poses a substantial issue in hydroelectric power, mining, petroleum refining, chemical processing, and aerospace sectors [1]. Components such as turbine blades, pump impellers, pipelines, and valves are often exposed to high-velocity fluid flows containing abrasive solid particulates [2]. These particles cause ongoing damage to surfaces, leading to weaker materials, shorter lifespans, more maintenance needs, and financial losses from unexpected shutdowns. Although filtering devices and separators are employed to reduce particle incursion, their total eradication is frequently unfeasible in practical applications [3–6]. Understanding slurry erosion is complicated because it depends on multiple interacting factors, such as the size and shape of the particles, how fast they hit, the angle they hit at, the concentration of the slurry, and the properties of the material being hit. Previous studies have also highlighted that impingement angle and surface interaction conditions significantly influence erosion mechanisms and surface damage behaviour under slurry erosion environments [7]. Moreover, turbulence in industrial fluid flows intensifies the complexity by unpredictably modifying the impact dynamics [8]. While initial research concentrated on assessing material degradation, later investigations prioritised the comprehension of particle-fluid-material interactions, microstructural damage, and removal procedures across various materials [9–13]. Experimental techniques are essential for characterising material behaviour under erosive circumstances. Field-scale testing, while precise in representing service circumstances, is expensive and inflexible [14–16]. Laboratory-scale rigs are favoured for their cost-effectiveness, parameter control, and capacity to expedite erosion processes. Among the numerous test rigs produced, including slurry pot [17], slinger [16], rotating arm [18], and jet-type rigs [19], the slurry jet erosion rig is distinguished by its operating flexibility and adaptability. Jet-type rigs allow for controlled settings for speed, impact angle, and slurry thickness, and can work with systems that recycle particles and those that do not. A significant problem in slurry erosion testing is the efficient incorporation of erosive particles into the fluid flow. Jet erosion rigs for slurry testing are categorised into pre-mixed, partially pre-mixed, and post-mixed systems based on the method of particle introduction [17,20,21].

**Fig 1:**
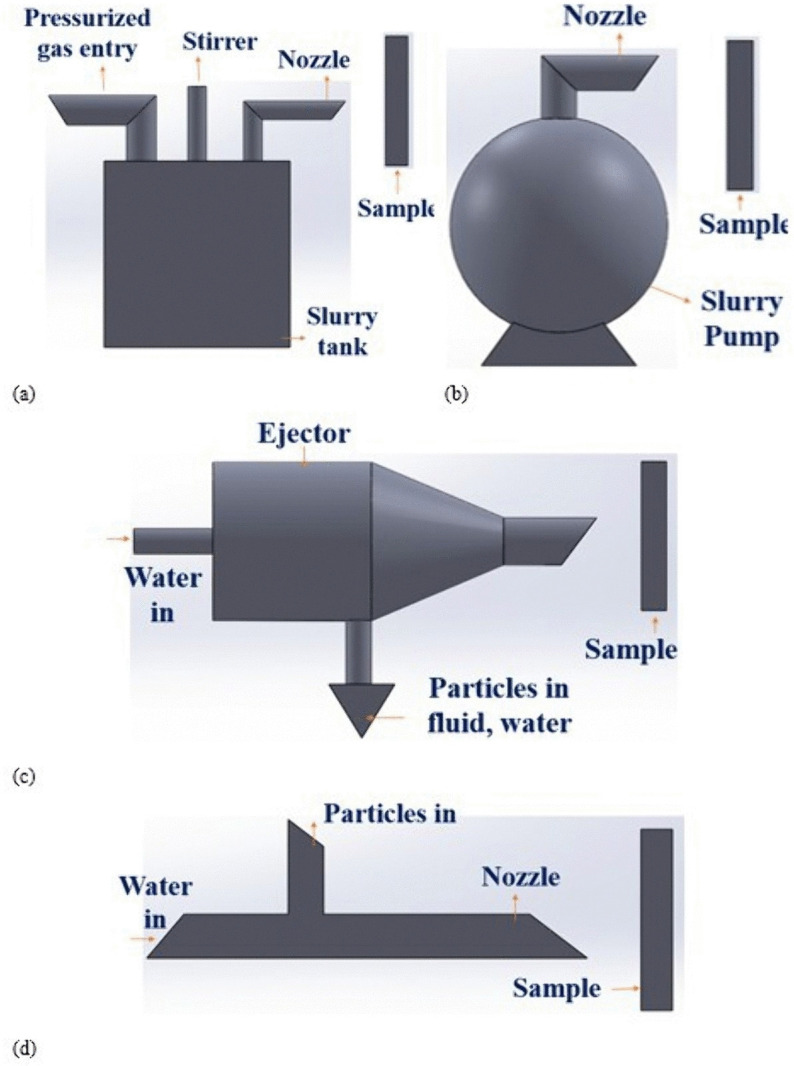
Classification of commonly used slurry jet erosion test configurations based on particle introduction mechanisms: (a) Levy method (pressurised premixed slurry system), (b) Turenne method (pump-driven premixed slurry system), (c) Hutchings method (Venturi-assisted partially premixed system), and (d) gravity-assisted post-mixed particle injection configuration adapted from previous slurry erosion test rig classifications.

Fig 1 presents the commonly employed slurry jet erosion test methodologies based on particle introduction mechanisms, including the pressurised premixed slurry configuration proposed by Levy [22], the pump-driven premixed system reported by Turenne [23], the Venturi-assisted partially premixed arrangement developed by Hutchings [18], and the gravity-assisted post-mixed particle injection configuration described in previous slurry erosion test rig classifications [24].

The different methods relate to specific ranges of parameters, as shown in Table 1, where velocity, particle concentration, erodent type, and size vary significantly across the test setups.

**Table 1:** Comparison of slurry erosion test rigs and their operating characteristics reported in literature.

Researchers	Velocity, m/s	Concentration, wt. %	Nozzle diameter (mm)	Erodent type	Erodent size (µm)	Ref.
Levy	9-38	10, 20, 30	3.125	Coal, Al_2_O_3_, SiC	24, 150	[17]
Turenne	17	1, 5, 10, 15, 20	4.76	Silica	200, 300	[23]
Hutchings	0-8	0-30	4.5-6.5	Silica	425-1000	[18]
Post-mixed configuration	50	0.28	8	River sand, Garnet	90-500	[24]

As contemporary researchers modified these methodologies, broader operational parameters were investigated. Table 2 presents an overview of the parameter ranges, indicating both the lower and upper bounds employed in previous experiments. While Table 1 compares the fundamental operating principles and configurations of different slurry erosion rigs, Table 2 summarizes the corresponding operating parameter ranges reported in earlier investigations.

**Table 2:** Operating parameter ranges adopted in previously reported slurry erosion studies.

Test method	Velocity, m/s		Concentration, wt. %		Particle size, µm		Ref.
	Low	High	Low	High	Low	High	
Levy	8	38	0.12	30	24	710	[22], [25,26]
Turenne	4	70	0.001	25	30	1190	[27–36]
Hutchings	1.5	11	0.1	40	50	1180	[37–40]
Post-mixed configuration	22	117.3	0.0015	0.4	150	500	[24,41–43]

Notwithstanding the diversity of slurry jet erosion test rigs and the documented range of operational parameters, a generally standardised test rig remains unavailable. This is mostly because of the interconnectedness of contributing elements and the difficulties in duplicating industrial flow conditions in a laboratory setting. Furthermore, challenges include particle recirculation, velocity regulation, erosion consistency, and the simplicity of particle injection persisting in current systems. This work presents a new Venturi-based setup for testing slurry jet erosion that offers a simpler and more effective way to inject particles using a modified venturimeter. This apparatus eliminates the need for intricate pressurisation systems or vacuum ejectors while enabling controlled regulation of slurry characteristics. To experimentally assess the operational capability of the developed rig, two representative metallic materials-brass (ductile) and cast iron (CI) (brittle)-were chosen. River sand with different particle sizes was used as the abrasive material, and important factors like the angle of impact and the concentration of the slurry were changed while keeping the speed the same. Despite the availability of several slurry jet erosion configurations, many existing systems rely on premixed slurry circulation, pressurised particle feeding, or auxiliary vacuum-assisted mechanisms that increase operational complexity and maintenance requirements. In addition, achieving stable particle entrainment, repeatable impact conditions, and controlled slurry delivery remains challenging in laboratory-scale erosion studies. The present work addresses these limitations through the development of a simplified Venturi-assisted particle entrainment system capable of introducing dry erosive particles directly into a flowing liquid stream under controlled operating conditions without the use of pressurised slurry chambers or external particle feeding systems.

Accordingly, the primary objective of the present investigation was to develop and experimentally evaluate a simplified Venturi-assisted slurry jet erosion test rig capable of controlled dry-particle entrainment under laboratory-scale conditions. A secondary objective was to assess the capability of the developed apparatus to distinguish erosion behaviour of representative ductile and brittle metallic materials under varying impingement angles, particle sizes, and slurry concentrations.

The experimental programme was therefore designed to evaluate the operational capability of the developed apparatus through comparative erosion responses rather than to provide comprehensive metallurgical or compositional characterisation of the test materials and erodent. Material composition was verified by optical emission spectroscopy, while pre- and post-erosion SEM observations were used qualitatively to support interpretation of the observed erosion mechanisms. The erodent was characterised in terms of particle-size distribution and surface morphology because these physical characteristics directly define the particle fractions and impact conditions employed in the erosion tests.

## 2 Materials and methods

### 2.1 Materials selection and characterization

In slurry erosion research, ductile materials generally display a characteristic wear pattern influenced by plastic deformation mechanisms. These encompass ploughing, micro-cutting, and extrusion of surface material, particularly under low-angle impact. This study used brass, a renowned copper-zinc alloy, to exemplify the ductile material group. Prior research [44,45] reveals that ductile materials often experience peak erosion at an impingement angle of 30°, making brass an appropriate option for evaluating the test rig's capacity to measure angle-dependent erosion behaviour. Its mild hardness and capacity for plastic deformation under repetitive particle impacts make it suitable for analysing erosive wear properties in metallic components used in valves, fittings, and low-pressure fluid systems.

CI was chosen as the representative brittle material due to its elevated carbon content, the presence of graphite flakes, and its intrinsic vulnerability to crack initiation under standard impact conditions. Brittle materials, in contrast to ductile ones, predominantly fail due to micro-cracking and fracture, particularly under direct (90°) impact. Previous studies have shown that brittle materials generally exhibit maximum erosion under normal or near-normal impact conditions because direct particle impact promotes crack initiation, brittle fracture, and material chipping [43,46,47]. Pumps, slurry transport pipes, and components exposed to abrasive conditions extensively utilize CI, thereby affirming its significance in this context.

The chemical compositions of both materials were examined using optical emission spectroscopy (OES) to verify compliance with industry-grade requirements. The materials were commercially procured, and compositional analysis was carried out using standard laboratory characterization procedures prior to slurry erosion testing. The results were consistent with the classification of brass as a ductile copper–zinc alloy and cast iron as a brittle ferrous alloy appropriate for comparative slurry erosion analysis. Tables 3 and 4 show the exact weight percentages of the added elements, explaining how they are expected to perform in environments that cause wear.

**Table 3:** Chemical composition of brass.

Element	Sn	Pb	Zn	Ni	P	Fe	Si	Mn	Al	Cu
Wt. %	0.41	3.98	36.72	0.32	0.003	0.40	0.02	0.03	0.24	Balance

**Table 4:** Chemical composition of CI.

Element	C	S	P	Si	Mn	Cu	Cr	Ni	Mo	Fe
Wt. %	3.04	0.11	0.068	2.58	0.42	0.05	0.07	0.02	0.005	Balance

**Fig 2:**
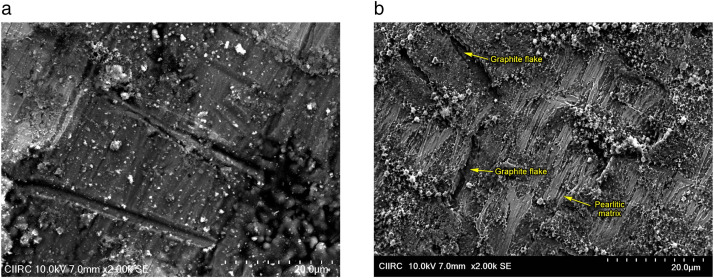
Representative SEM images of (a) brass and (b) grey cast iron specimens prior to slurry erosion testing.

The brass and cast iron specimens used in the present investigation were prepared as rectangular samples of dimensions 25 mm × 25 mm × 6 mm. Prior to erosion testing, the specimen surfaces were prepared to obtain consistent initial surface conditions for comparative erosion assessment. Representative SEM images of the investigated materials prior to slurry erosion testing are presented in Fig 2. These images were used for qualitative examination of the representative initial surface features before erosion testing.

#### 2.1.1 Initial material characterisation.

Representative pre-erosion SEM images of brass and grey cast iron are presented in Fig 2. The images were examined qualitatively to document representative initial surface features of the specimens before slurry erosion testing. The brass specimen exhibited a comparatively uniform surface appearance, whereas the grey cast iron specimen showed heterogeneous microstructural features consistent with the contrasting material characteristics of the two investigated alloys. The purpose of the pre-erosion examination was to establish a qualitative reference condition for comparison with the erosion-induced surface morphologies discussed subsequently. Detailed phase identification and localized elemental analysis were beyond the scope of the present investigation. The bulk chemical compositions of both materials were independently determined using optical emission spectroscopy and are reported in Tables 3 and 4.

### 2.2 Design of the slurry jet erosion test rig

#### 2.2.1 Limitations of conventional designs.

Traditional slurry erosion apparatus frequently utilizes mechanisms such as pressurized tanks, premixed slurry pumps, or vacuum ejectors to transport erosive particles to a designated specimen. These setups, while effective, have many limitations, such as inconsistent flow, difficulties in managing factors like particle concentration or impact angle, and maintenance issues due to slurry sedimentation, particle settling, and flow instability during prolonged operation. Furthermore, certain designs necessitate extensive infrastructure and expensive control systems, rendering them inappropriate for adaptable laboratory-scale research.

#### 2.2.2 Development of the modified venturi device.

A novel Venturi-based slurry jet erosion apparatus was created to overcome these constraints. The design is based on the Venturi principle, in which reduction in flow cross-sectional area increases fluid velocity while simultaneously reducing static pressure at the throat section. This low-pressure area is deliberately employed to inject dry erosive particles into the water flow via a funnel. The diverging segment of the Venturimeter was eliminated to decrease flow disruption, lessen pump load, and provide a concentrated impact stream on the specimen surface.

#### 2.2.3 Schematic description and working principle.

**Fig 3:**
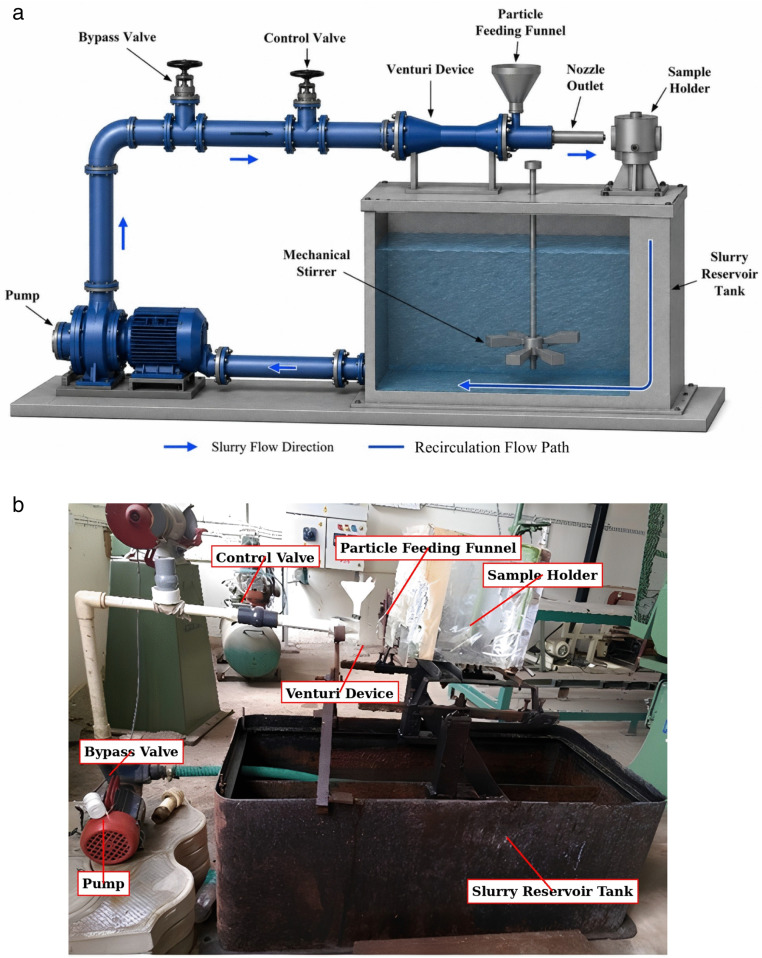
Developed Venturi-based slurry jet erosion test rig showing (a) schematic 3D representation of the slurry circulation path, Venturi section, particle feeding arrangement, nozzle, and specimen holder, and (b) photograph of the laboratory-scale apparatus with major components identified.

Fig 3(a) illustrates the schematic configuration and working principle of the developed apparatus, whereas Fig 3(b) presents the actual laboratory-scale experimental setup employed for slurry erosion testing. The setup consists of a water reservoir tank, a centrifugal pump for slurry circulation, a bypass valve for flow regulation, a Venturi section integrated with a particle feeding funnel, and a specimen holder positioned near the nozzle outlet. During operation, the pump drives the fluid through the converging section of the Venturi assembly, generating a localized pressure reduction that assists particle entrainment into the flowing stream prior to impingement on the test specimen. As velocity escalates and pressure diminishes at the throat, dry particles descend from the funnel into the stream and are entrained towards the specimen. The impingement angle was adjusted by repositioning the specimen holder relative to the nozzle exit. This design enables controlled regulation of factors including flow velocity, particle concentration, and impact angle without the necessity for intricate electronics or vacuum systems. The Venturi-based configuration was selected because it enables passive particle entrainment without pressurised slurry circulation systems, thereby reducing flow instability, maintenance complexity, and equipment cost compared with conventional premixed-slurry jet erosion rigs.

The external corrosion visible on the reservoir tank in the laboratory photograph is attributable to prolonged exposure to water during routine operation and storage of the experimental apparatus. The visible corrosion was limited to the external reservoir surface and resulted from long-term exposure to water during routine laboratory operation. No internal degradation affecting slurry circulation, particle entrainment, flow regulation, or erosion measurements was observed during testing. The Venturi section consisted of a converging nozzle with an inlet diameter of 25 mm and a throat diameter of 8 mm. The specimen was positioned at a fixed stand-off distance of 20 mm from the nozzle exit. A single-stage centrifugal pump with a rated discharge capacity of 30 L/min was used to circulate water through the system. The bypass valve was incorporated to regulate discharge flow rate and maintain stable flow conditions during experimentation, while the secondary control valve was used for isolation and flow adjustment during system operation and cleaning.

The developed setup operates as a recirculating slurry erosion system in which erodent particles, after impingement, are collected in the reservoir and re-entrained into the flow stream. Continuous stirring inside the reservoir was maintained to minimise particle settling and promote uniform slurry distribution throughout the experimental duration. The Venturi-assisted configuration facilitated continuous particle entrainment near the low-pressure throat region and enabled controlled slurry delivery during laboratory-scale testing. Continuous stirring inside the reservoir minimised sedimentation during recirculation, while the bypass valve arrangement enabled stable discharge regulation throughout testing. The specimen holder geometry permitted controlled adjustment of impingement angle and stand-off distance during experimentation. No evidence of cavitation-related noise, vibration, or operational instability was observed during testing at the selected laboratory-scale operating velocity of 6 m/s.

### 2.3 Experimental procedure

#### 2.3.1 Test standards and protocol.

The slurry erosion experiments were conducted following operational principles adapted from ASTM G73 for controlled liquid-jet erosion testing, ensuring consistent regulation of velocity, impingement angle, and exposure duration during experimentation [48]. The repeatability of erosion measurements under identical operating conditions indicated reasonably stable particle entrainment and repeatable impact behaviour throughout the experimental programme. Each experimental condition was repeated three times, and the average values were reported to minimise random experimental variability. The fluid velocity was determined using the volumetric method, which involved recording the time required to fill a container of known volume to calculate the discharge rate according to the continuity equation. This method, endorsed in previous research [18,40], is broadly recognized in erosion testing for its simplicity and practical reliability; and requires no advanced instrumentation.

#### 2.3.2 Test parameters and setup.

The slurry concentration was controlled by varying the mass flow rate of dry erodent particles introduced through the feeding funnel while maintaining constant water discharge conditions. The required slurry concentration levels were established using pre-measured quantities of erodent particles introduced during the selected test duration. Prior to each experiment, the required mass of river sand corresponding to the target slurry concentration was measured using a digital balance and introduced into a known volume of water. No additional particles were added during testing. Continuous mechanical stirring was maintained throughout the experiment to minimize particle settling and maintain approximately uniform particle dispersion within the recirculating slurry system. Flow velocity was adjusted using the bypass valve arrangement and verified experimentally through volumetric discharge measurements. The desired slurry concentration was established by introducing a predetermined mass of erodent particles corresponding to the selected concentration (0.1 or 0.2 wt.%) into the recirculating water volume prior to testing. During operation, the particles recirculated within the closed-loop system and were continuously suspended by mechanical stirring to maintain approximately uniform concentration throughout the test duration. The feeding funnel served as the particle entrainment point into the Venturi stream, whereas the overall slurry concentration was governed by the initial particle mass-to-water ratio. The experiment was conducted at a constant water velocity of 6 m/s. Two slurry concentrations, 0.1 wt.% and 0.2 wt.%, were employed to investigate the impact of erosive particle loading. The impingement angles varied from 15^°^ to 90^°^ degrees, in increments of 15^°^, to examine angle-dependent erosion patterns. The erosive particles comprised river sand with specified sizes of 360 µm, 510 µm, and 655 µm, determined using sieve analysis, as described in the particle characterization procedure. Each test was performed for a duration of 10 or 30 minutes, with specimen weight recorded at 5-minute intervals to get high-resolution temporal data. Table 5 gives a detailed summary of the experimental matrix.

**Table 5:** Test parameters adopted in the investigation.

Parameter	Value
Velocity of fluid (m/s)	6 (constant)
Slurry concentration (wt.%)	0.1, 0.2
Erosive particle type	River sand
Particle sizes used (µm)	360, 510, 655
Impingement angles (°)	15° to 90° (intervals of 15°)
Test durations (minutes)	10, 30 (interrupted at 5-min steps)

The erosion rate was calculated based on the actual projected eroded area measured from the visible wear scar region rather than the total specimen surface area. For elliptical erosion scars formed at lower impingement angles, the projected eroded area was calculated using (A = \pi ab), where (a) and (b) represent the semi-major and semi-minor axes of the scar, respectively. For near-circular scars observed under normal impingement conditions, the projected area was calculated using (A = \pi r^2), where (r) is the scar radius. The scar dimensions were measured from optical images, and the calculated projected area was used to normalize weight loss values and determine erosion rates (mg/mm^2^).

#### 2.3.3 Surface roughness and hardness measurement.

To understand how erosion affects strength, Vickers microhardness tests were performed on both materials before testing in accordance with ASTM E384 using a load of 1000 g applied for 30s [49]. Microhardness measurements were obtained at three different locations on each polished specimen surface, and the average value was reported to minimise the influence of local surface heterogeneity. Surface roughness was assessed using a Mitutoyo Talysurf surface profilometer before and after each test to quantify the changes in surface texture resulting from particle impingement. These assessments offered insight into microstructural degradation and possible loci for fatigue or crack development.

#### 2.3.4 Erodent particle characterisation and SEM morphology analysis.

To guarantee uniform and replicable erosion behaviour, the river sand employed as an erosive medium was analysed for particle size distribution with a vibratory sieve analyzer. The sand was dehydrated in an oven and subjected to sieving for 15 minutes through seven distinct mesh sizes, varying from 1180 µm to less than 180 µm. The mass of material retained on each filter was documented to ascertain the distribution and identify predominant size fractions.

**Fig 4:**
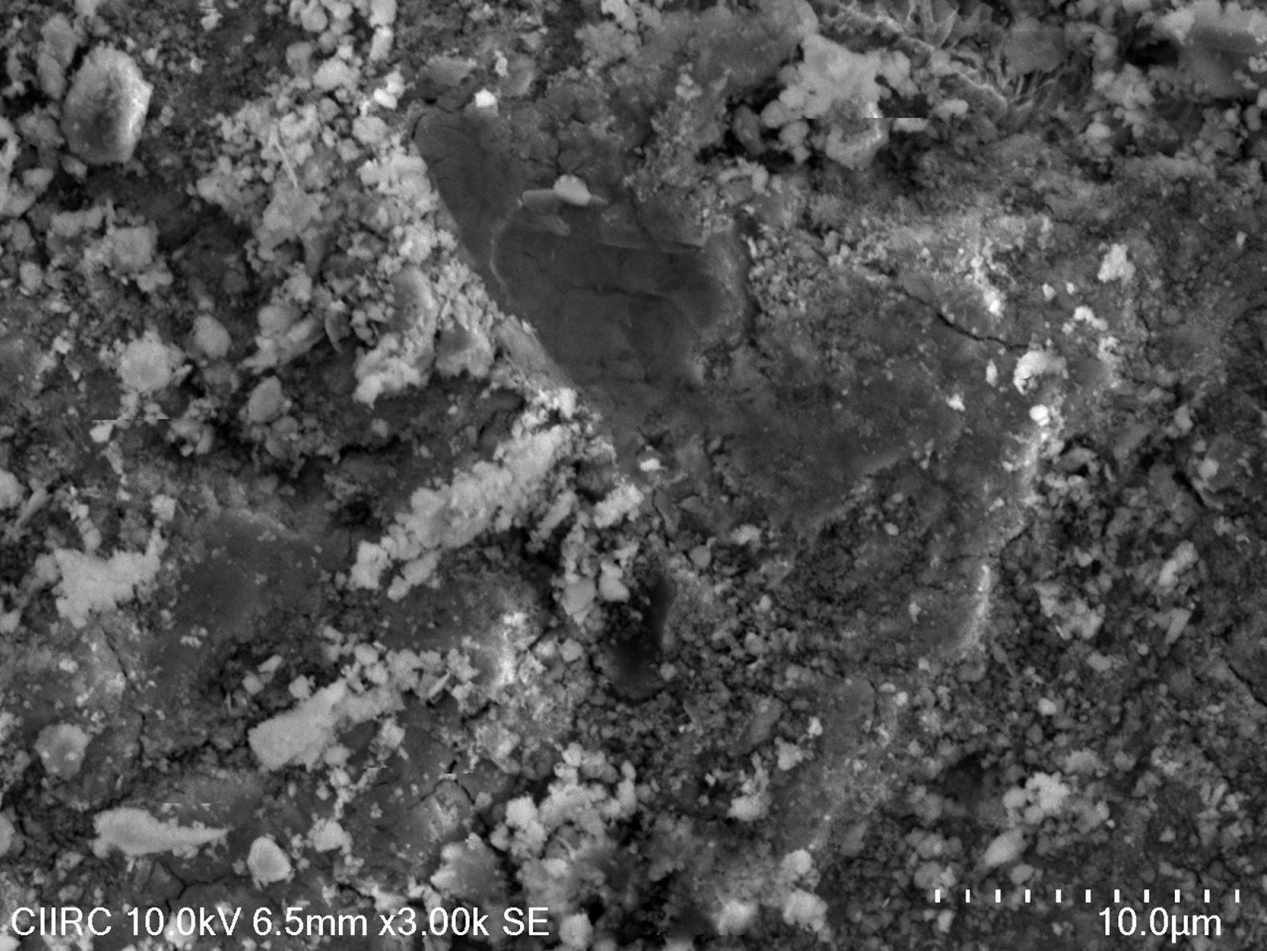
Representative SEM morphology of the river sand erodent showing the irregular and angular particle characteristics considered in the slurry erosion experiments.

Three principal size categories – 360 µm, 510 µm, and 655 µm were chosen for testing based on the sieve results. The selected sizes were intended to replicate actual slurry conditions and to assess the influence of augmented particle size on erosion intensity. This classification facilitated the investigation of kinetic energy under regulated velocity settings. Representative SEM morphology of the river sand particles used as erodent in the slurry erosion experiments is shown in Fig 4. The particles exhibited irregular, angular geometries, rough surfaces, and non-uniform edges characteristic of naturally occurring river sand. These morphological characteristics are relevant to slurry erosion because angular edges and irregular particle geometries promote localized stress concentration, cutting, ploughing, and impact-induced surface damage during particle–material interaction. The SEM examination was therefore used to establish the physical morphology of the erodent employed in the experiments.

The observed morphology supported the suitability of the selected river sand particles for laboratory-scale slurry erosion investigations. The erosive potential of particles is closely linked to their kinetic energy, which is a function of mass and velocity. The kinetic energy estimation was approximated using an equivalent spherical particle assumption, and the particle impact energy was calculated using the following relation [50]:


$$\begin{array}{c} KE=\frac{\text{1}}{\text{2}}mv^{\text{2}} \end{array}$$ (1)

where *KE* is kinetic energy; *m* is mass of the erosive particle; *v* is velocity.

Assuming spherical geometry, the mass *m* is derived from volume and density:


$$\begin{array}{c} V=\frac{\text{4}}{\text{3}}\pi {r_{p}}^{\text{3}} \end{array}$$ (2)

where *V* is volume; *r*_*p*_ is radius of the particle.

Substituting this into the kinetic energy formula yields the final expression used [51]:


$$\begin{array}{c} KE=\frac{\text{2}}{\text{3}}\pi \rho_{p}{r_{p}}^{\text{3}}v^{\text{2}} \end{array}$$ (3)

where *ρ*_*p*_ is density of the particle.

This calculation, adapted from [46], enabled estimation of the minimum energy threshold required for erosion initiation in each material, thereby helping correlate theoretical predictions with experimentally observed erosion behaviour. Although the erosive particles possessed irregular angular morphology, the equivalent spherical particle assumption was adopted only for approximate kinetic energy estimation and comparative threshold analysis, consistent with simplified approaches commonly employed in slurry erosion studies.

## 3 Results and discussion

### 3.1 Hardness and surface roughness analysis

**Fig 5:**
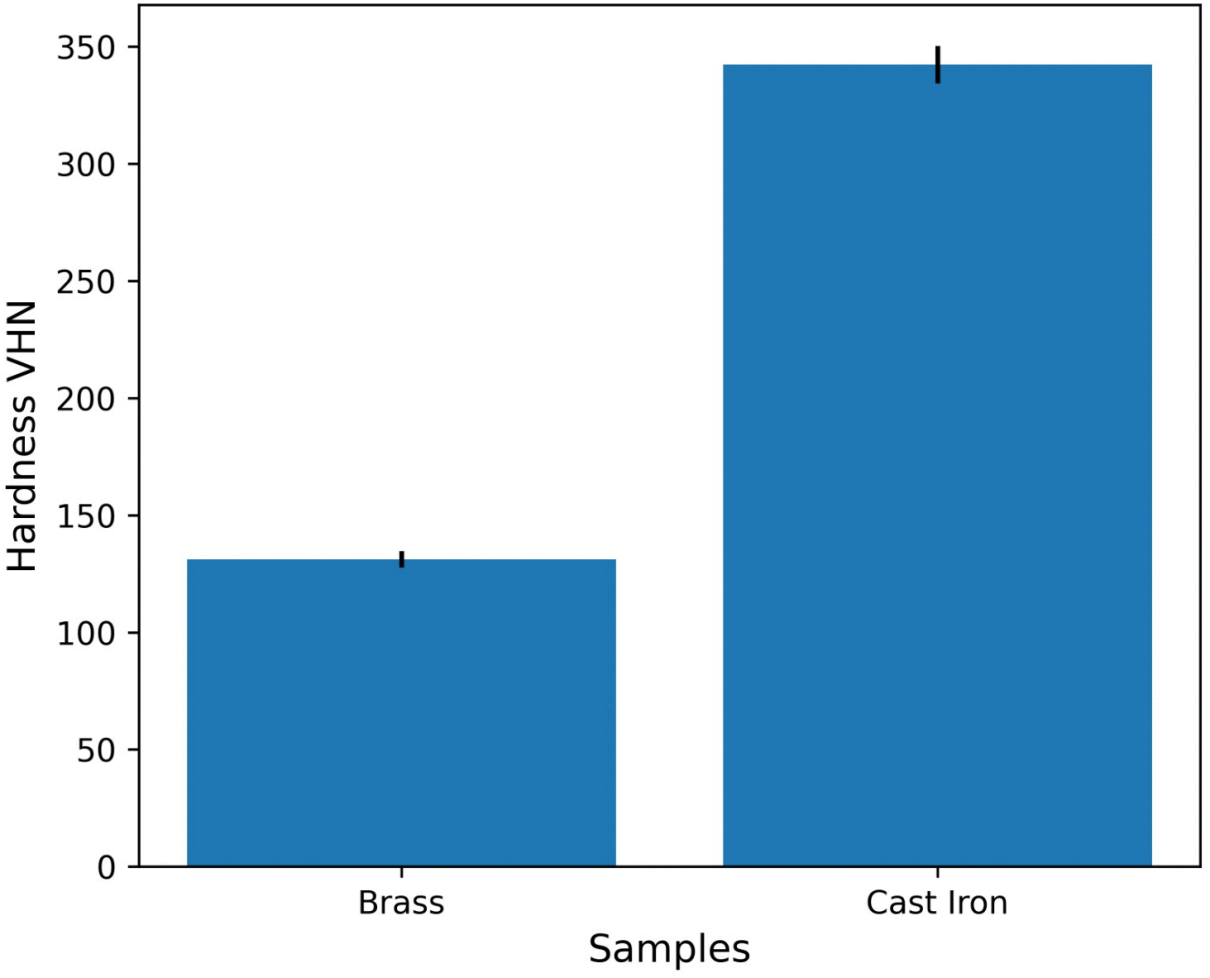
Comparison of Vickers micro hardness values for brass and CI used in the slurry erosion test.

The hardness of a material is a crucial mechanical attribute affecting its resistance to erosive wear. Hardness significantly influences resistance to particle indentation and surface deformation during erosive impact conditions. Fig 5 demonstrates that the Vickers Hardness Number (VHN) for brass is 131.2, while CI displays a markedly elevated hardness value of 342.3. The variation in hardness measurements across the tested locations remained within ±5% of the reported average values, indicating acceptable measurement consistency and surface uniformity for comparative erosion analysis. The pronounced difference in hardness is attributed to the distinct microstructures of the two materials. Brass, being a ductile alloy, possesses a comparatively softer copper–zinc matrix that facilitates plastic deformation under erosive impact. In contrast, the higher measured hardness of CI provides greater resistance to surface indentation, while its comparatively brittle material response promotes crack initiation and fracture during particle impingement.

The ramifications of this disparity in hardness are especially pertinent in slurry erosion tests. The higher hardness of CI means it can better resist damage from small particles hitting its surface, especially when they are moving slowly. However, as explained in the following sections, the brittle characteristics of CI can lead to material removal mechanisms caused by fractures under specific impact conditions.

### 3.2 Effect of particle size

**Fig 6:**
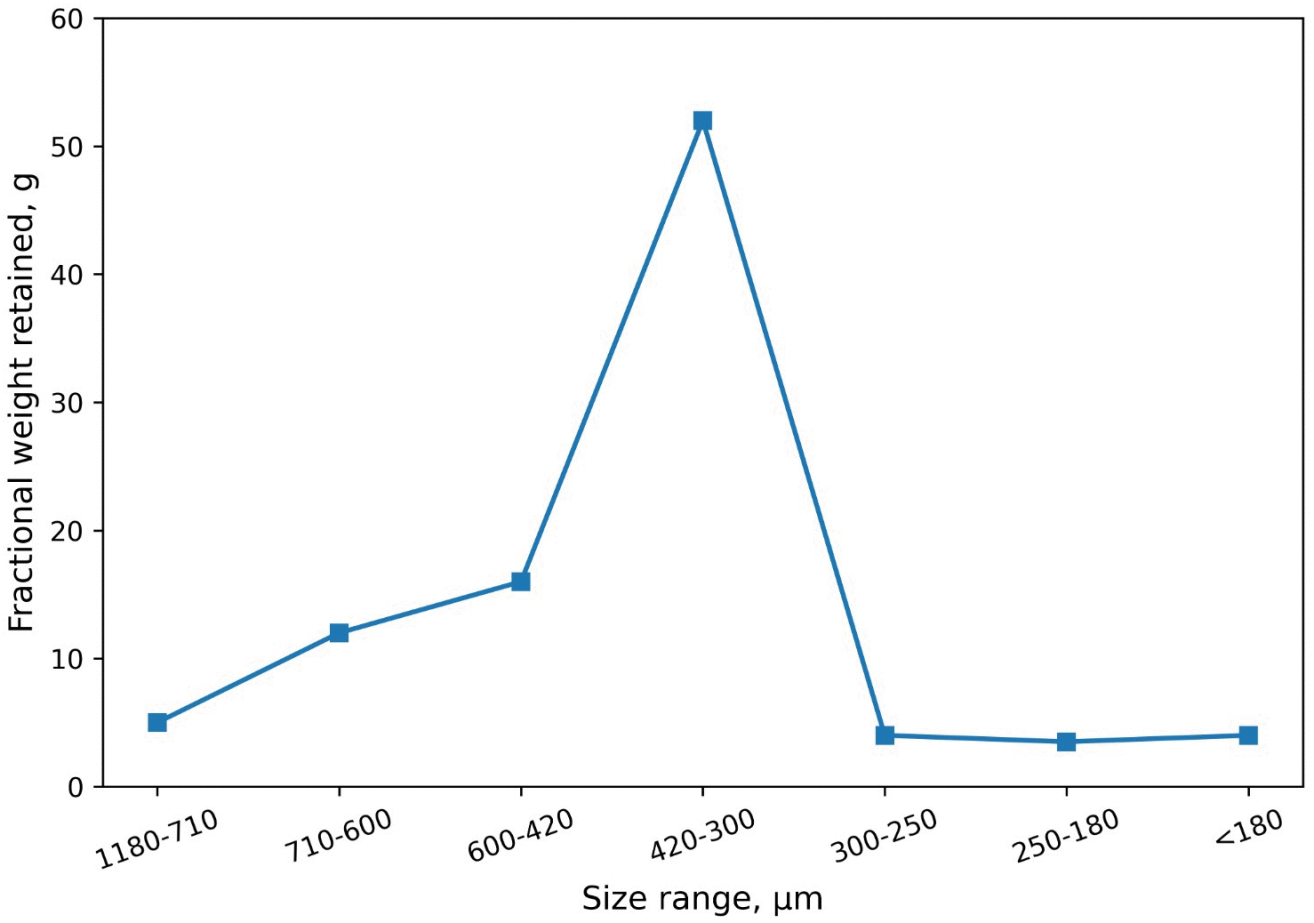
Particle size distribution of river sand based on vibratory sieve analysis.

The size, shape, and spread of solid particles greatly affect how much they can wear away surfaces in slurry erosion studies. A detailed study of the particle size distribution of the river sand used for erosion tests was carried out with a vibratory sieve analyser to maintain consistency and experimental control in the experiments. The dried sand was subjected to sieving through a sequence of conventional sieves corresponding to the following particle size ranges: 1180−710 µm, 710−600 µm, 600−420 µm, 420−300 µm, 300−250 µm, 250−180 µm, and below 180 µm. The mass collected in each filter was documented to ascertain the proportion of each size category. The distribution curve depicted in Fig 6 indicates that the predominant particle fraction is within the 420−300 µm range, accounting for almost 50% of the total weight of the sand sample. The result indicates that particles within this range are predominant in the erosive environment and are therefore anticipated to have a substantial role in the erosion process.

Based on the sieve analysis results, extremely large and very fine particle fractions, specifically those within the ranges of 1180–710 µm, 300–250 µm, 250–180 µm, and <180 µm, were excluded from subsequent erosion experiments. The chosen particle sizes for erosion testing were 360 µm, 510 µm, and 655 µm, obtained from the mid-ranges of the retained fractions.This selection facilitates the examination of size-dependent erosion behaviour while maintaining an accurate depiction of particles, as observed in hydraulic and abrasive slurry systems. The irregular angular morphology of the river sand particles provides realistic simulation of erosive conditions encountered in hydraulic slurry transport systems compared with idealised spherical abrasives. Although the erosive particles possessed irregular angular morphology, the equivalent spherical particle assumption was adopted only for approximate kinetic energy estimation and comparative threshold analysis, consistent with simplified approaches commonly used in slurry erosion studies.

**Fig 7:**
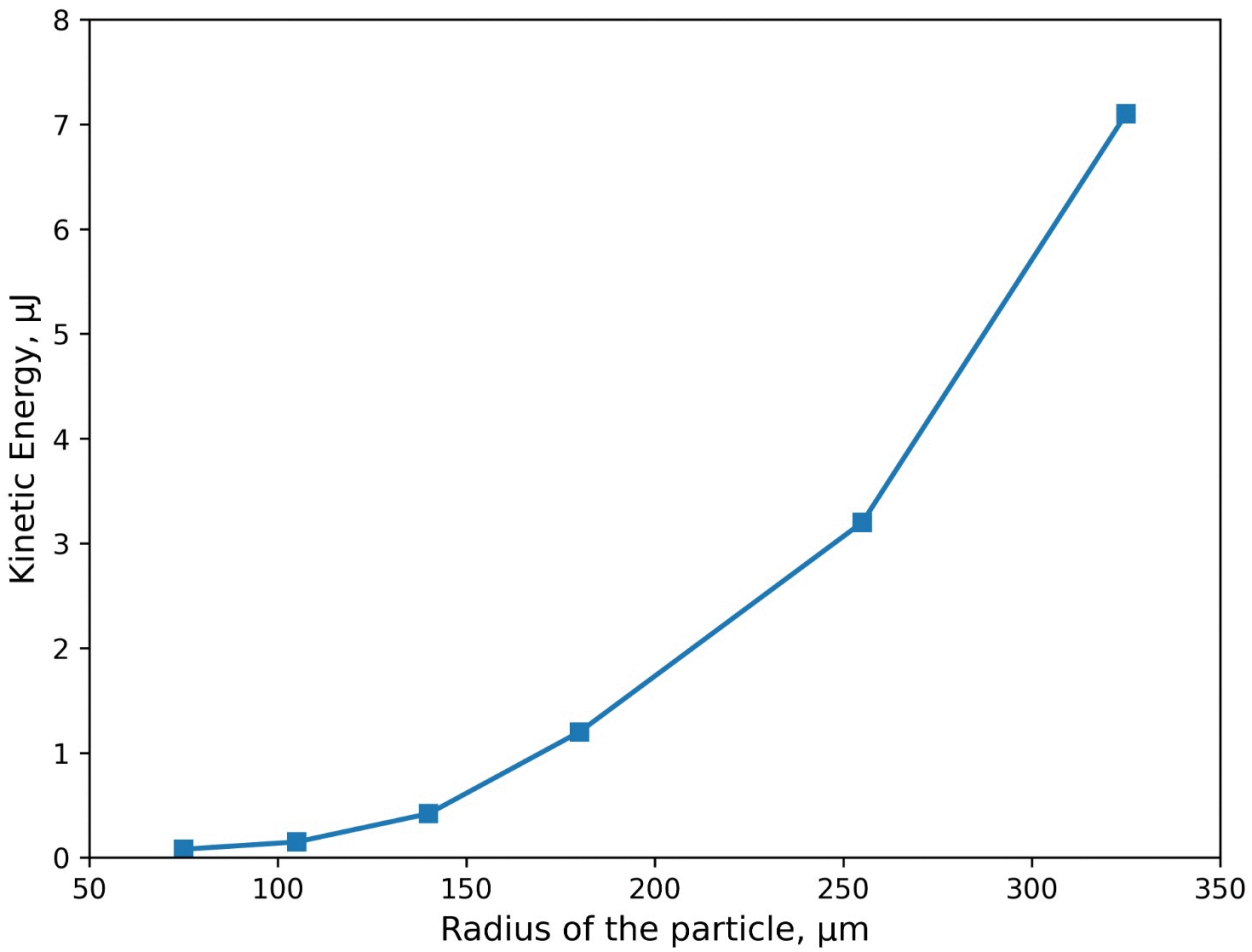
Kinetic energy variation with particle radius at a constant flow velocity of 6 m/s.

The kinetic energy of particles in slurry erosion processes markedly affects the extent and initiation of material degradation. The energy delivered by an impacting particle at a constant velocity escalates with its size, according to the cubic correlation between particle radius and mass. This characteristic is particularly crucial for assessing materials with differing erosion thresholds, such as ductile and brittle materials. This study estimated kinetic energy estimates for particles with diameters from 75 µm to 710 µm at a constant flow velocity of 6 m/s. Fig 7 illustrates a significant rise in kinetic energy with particle radius, escalating from below 0.1 µJ to above 7 µJ. This rapid increase shows that larger particles can cause much more erosion, even when the flow conditions stay the same.

**Fig 8:**
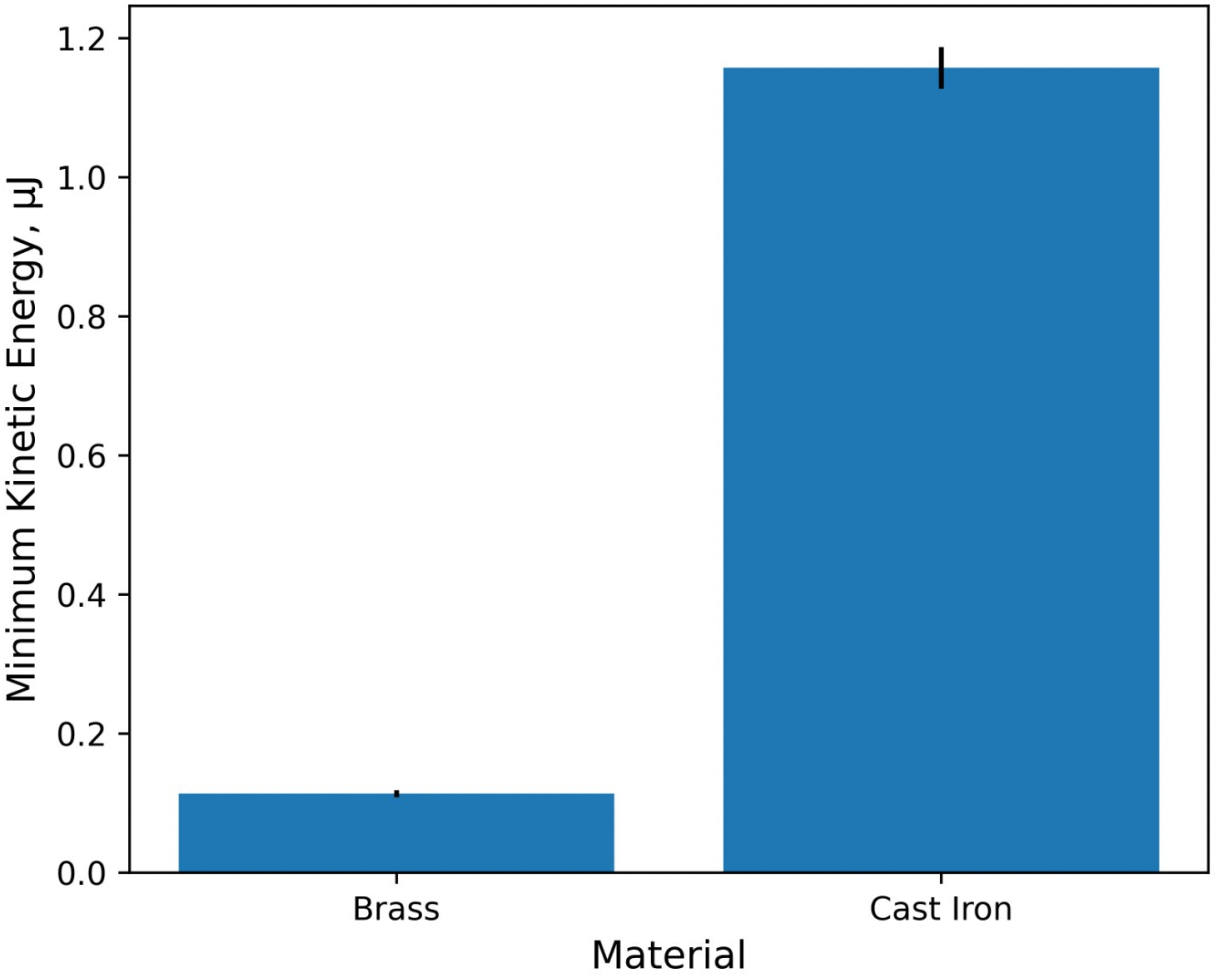
Minimum kinetic energy required to initiate erosion in Brass and CI specimens as identified in pilot trials.

A pilot-scale erosion study was undertaken to evaluate the threshold energy necessary to commence erosion on the test materials, utilising particle sizes ranging from greater than 75 µm to 710 µm. For brass, significant erosion commenced at a particle diameter of 165 µm, correlating to a kinetic energy of roughly 0.1135 µJ. This finding matches what is already known, which shows that ductile materials can wear away with low-energy impacts because of processes like localized plastic deformation and surface shearing. Conversely, cast iron, characterised by its brittle behaviour, demonstrated no material loss for particle sizes under 300 µm, even after numerous erosion test cycles. Erosive wear on CI became apparent only with particles above 300 µm, where the corresponding kinetic energy surpassed 1.1572 µJ. Fig 8 clearly illustrates the disparity in minimum kinetic energy limits between brass and CI. This notable contrast indicates that brittle materials such as CI necessitate greater impact energy to induce surface cracking, fragmentation, or chipping [52].

Consequent to these findings, particle sizes of 360 µm, 510 µm, and 655 µm were used for the ensuing experimental trials. The sizes conformed to the predominant fractions identified in the particle size distribution (as discussed in the particle size distribution analysis) and ensured that the kinetic energy imparted during tests significantly exceeded the minimal threshold necessary to provoke erosion in both brass and CI. This methodology facilitates uniform and significant comparisons among material varieties and enhances the integrity of the experimental design.

### 3.3 Effect of slurry concentration

The efficacy of the constructed slurry jet erosion test apparatus was assessed by examining the erosion properties of brass and CI under diverse operational settings. These tests elucidate the influence of material type, test duration, impingement angle, particle size, and slurry concentration on erosion patterns. The findings are summarised and analysed below. Each erosion test condition was repeated under identical operating parameters to ensure measurement consistency, and the reported values represent averaged observations obtained from repeated experimental trials.

**Fig 9:**
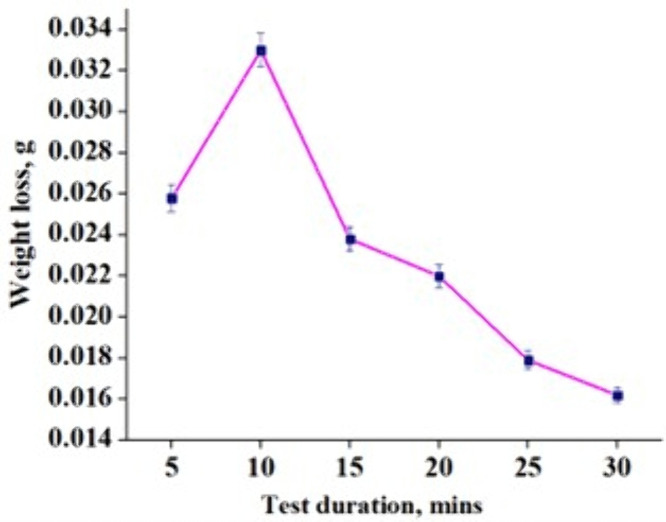
Weight loss of Brass vs. test duration (30 minutes).

**Fig 10:**
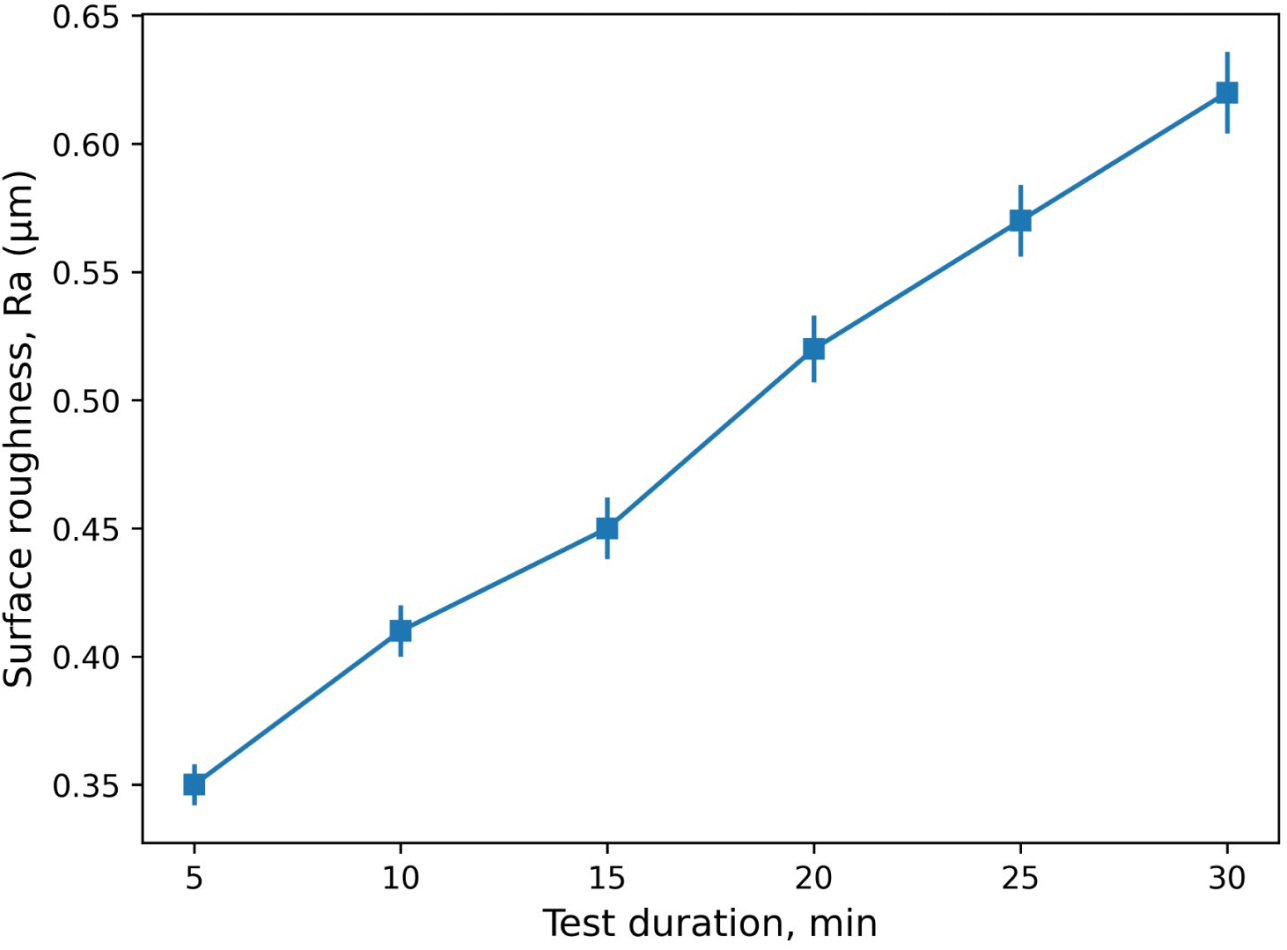
Surface roughness of Brass vs. test duration (30 minutes).

Brass was selected as a representative ductile material for slurry erosion evaluation [53]. During the initial step of the experiment, brass specimens underwent slurry erosion at a 30° impingement angle, which is known to induce maximal erosion in ductile materials [47]. The slurry concentration was maintained at 0.2 wt.%, and the testing period was prolonged to 30 minutes, with pauses every 5 minutes to evaluate incremental material loss. As illustrated in Fig 9, weight loss initially escalated and reached a maximum at the 10-minute interval. The subsequent reduction in erosion rate is due to the material's surface becoming harder, which happens when it gets hit repeatedly, making it tougher and less likely to lose more material. These results align with findings from prior erosion research on ductile metals [54]. Supplementary surface roughness measurements (Fig 10) indicate a consistent rise in roughness throughout the test. This behaviour, despite a reduction in weight loss, indicates that the surface continues to undergo micro-cutting and pitting, even as bulk removal diminishes. A coarser surface subjected to repetitive impact may exhibit increased vulnerability to fatigue and crack development, indicating a risk of long-term failure.

### 3.4 Effect of impingement angle

**Fig 11:**
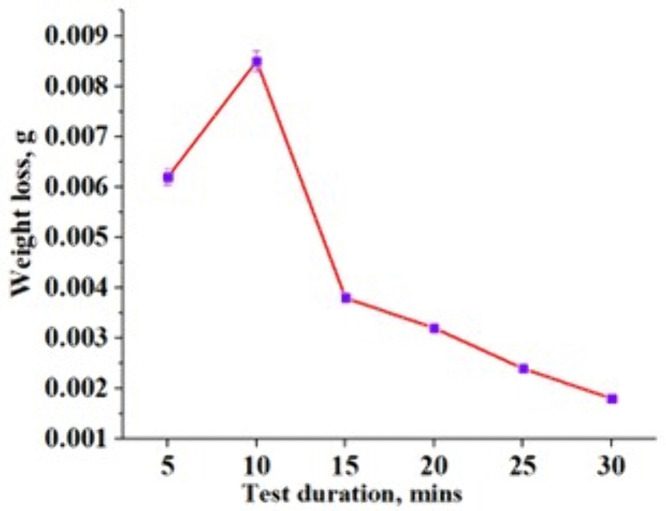
Weight loss of CI vs. test duration (30 minutes).

**Fig 12:**
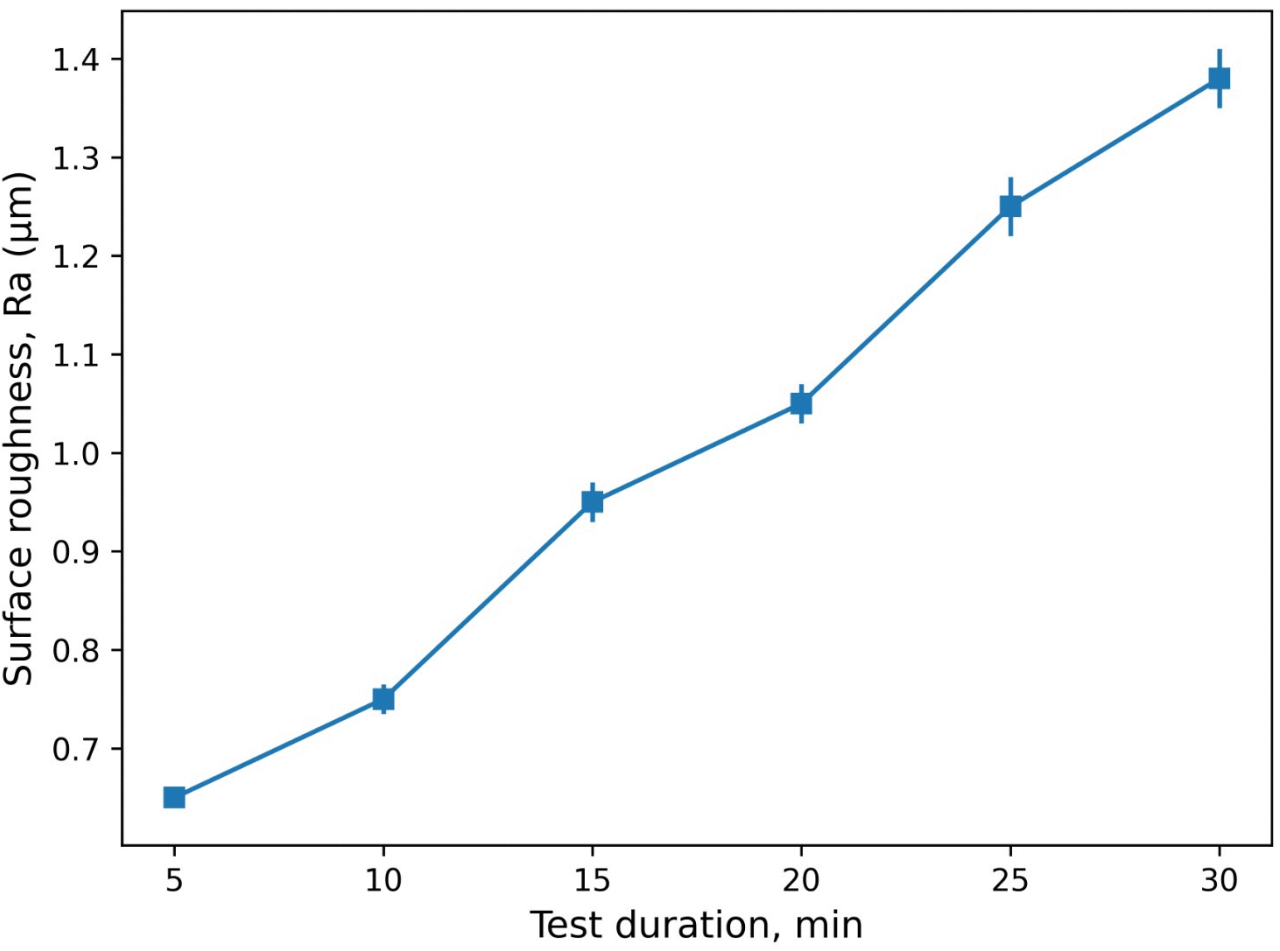
Surface roughness of CI vs. test duration (30 minutes).

A similar experimental procedure was conducted for CI specimens at a 90° impingement angle, consistent with previous studies reporting maximum erosion in brittle materials under normal impact conditions [44,52]. Fig 11 depicts the erosion trend of CI over 30 minutes. Like brass, CI exhibited an initial increase in weight loss, reaching a maximum at 10 minutes, followed by a gradual decline thereafter. This behaviour indicates transition from initial surface fracture initiation to stabilised erosion dominated by crack propagation and fragment detachment mechanisms. However, the total weight loss was lower than that of brass under the investigated conditions, which may be associated with the substantially higher measured hardness of CI and its greater resistance to erosive indentation. The surface roughness trends depicted in Fig 12 exhibit a significantly steeper increase relative to brass. The rigid yet fragile characteristics of CI cause surface fractures and chipping instead of plastic deformation, resulting in the emergence of sharp edges and deeper cavities. This observation corresponds with the erosion characteristics of brittle materials recorded in the literature [13] and verifies that surface damage is more pronounced in morphology but not necessarily in total material removal. The results from this test series demonstrated that a 10-minute timeframe is adequate to capture typical erosion behaviour for both materials under diverse circumstances.

**Fig 13:**
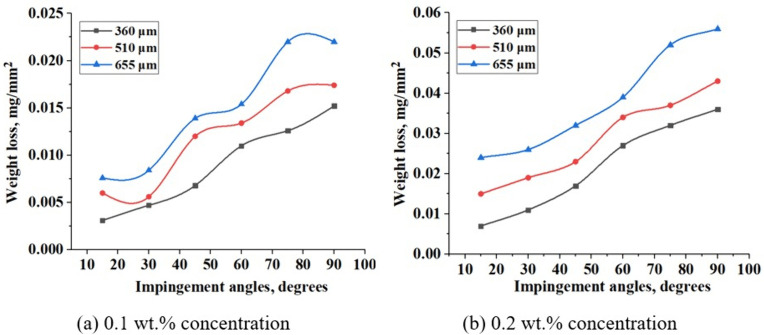
Slurry erosion of Brass at different impingement angles and particle sizes.

The study examined the effects of particle size and impingement angle at varying slurry concentrations, utilising erosion tests on both materials with three particle sizes (360 µm, 510 µm, and 655 µm) at two concentrations (0.1 wt.% and 0.2 wt.%). Relatively low slurry concentrations were selected during the preliminary laboratory-scale evaluation stage to maintain controlled particle entrainment while minimizing excessive particle agglomeration, sedimentation, and flow instability within the recirculating slurry system. The selected concentration range also enabled clearer assessment of material-dependent erosion trends under controlled operating conditions. The impingement angle was altered from 15^°^ to 90^°^, and the erosion rate was measured as weight loss per unit area (mg/mm²). Erosion for brass was continuously maximum at 30°, irrespective of particle size or concentration. This observation is consistent with the characteristic erosion response of ductile materials, wherein shallow-angle impacts induce considerable plastic deformation and surface abrasion [47]. Furthermore, it was apparent that bigger particles induced increased erosion owing to their elevated kinetic energy [12]. Similar trends associated with the influence of particle size and material matrix on erosion severity have also been reported in earlier investigations [55]. An inconspicuous anomaly was seen in Fig 13, indicating that the 655 µm particles exhibited reduced weight loss compared to the 510 µm particles at elevated angles. The difference may result from momentum dispersion or rebounds at greater sizes, which diminish effective energy transmission. At higher impingement angles, larger particles may experience partial momentum dissipation and rebound effects during impact, thereby reducing the effective transfer of kinetic energy to the target surface despite increased particle mass [29,52].

**Fig 14:**
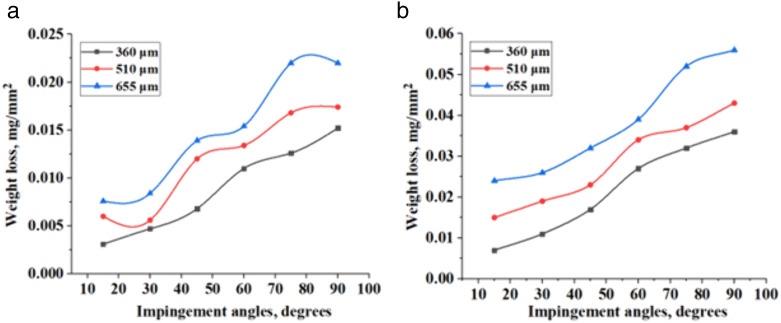
Slurry erosion of CI at different impingement angles and particle sizes.

The erosion behaviour of CI exhibited an inverse trend. Fig 14(a) and 14(b) show that the most weight was lost at a 90^°^ angle, which suggests that the erosion was mainly caused by small fractures and cracks forming in the material [44]. As the angle grew, especially at normal incidence, both the depth and severity of cracks escalated, resulting in more weight loss. Larger particle size and concentration enhance erosion due to increased impact energy and particle load.

In summary, brass exhibited greater total material removal than CI under comparable conditions. This behaviour may be associated with the lower measured hardness of brass, which renders it more susceptible to plastic deformation and progressive material removal. Conversely, CI exhibited lower material removal under the investigated conditions, which may be associated with its higher measured hardness; however, its comparatively brittle response resulted in surface roughening and localised chipping during erosive impact.

### 3.5 Wear scar analysis

**Fig 15:**
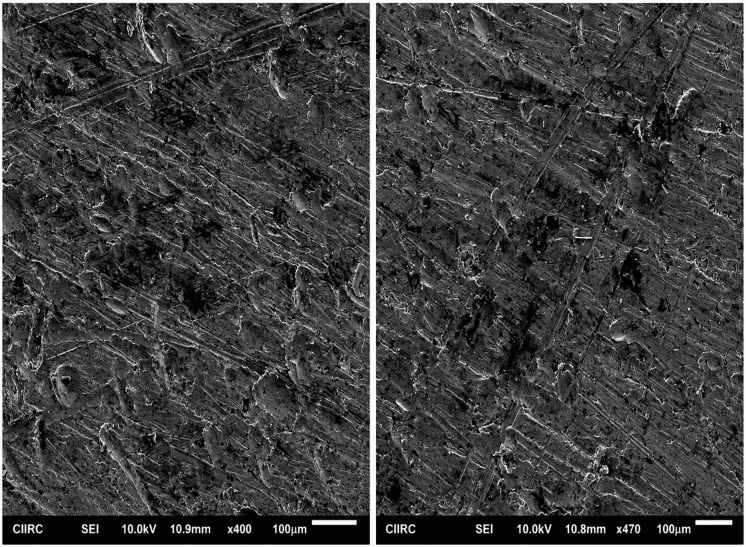
Representative wear scar morphologies observed after slurry erosion testing: (a) brass specimen showing an elongated erosion scar associated with ductile erosion behaviour and (b) cast iron specimen showing a localised erosion scar associated with brittle erosion behaviour.

Optical examination of the eroded specimen surfaces revealed distinct wear scar morphologies corresponding to ductile and brittle erosion behaviour. Brass specimens tested at lower impingement angles exhibited elongated elliptical scars aligned with the slurry flow direction, indicating dominant micro-cutting and ploughing mechanisms. In contrast, cast iron specimens subjected to near-normal impingement angles displayed relatively circular and localised erosion scars associated with brittle fracture and crater formation. The observed scar geometries were consistent with the measured erosion trends and SEM-based surface morphology analysis. Representative macroscopic wear scar morphologies developed under characteristic ductile and brittle erosion conditions are presented in Fig 15.

The elongated elliptical wear scar observed on brass at low impingement angle confirms the predominance of sliding abrasion, micro-cutting, and ploughing mechanisms caused by tangential particle motion. In contrast, the comparatively circular scar formed on cast iron under normal impact conditions indicates localized brittle fracture and concentrated particle impingement. The erosion rate calculations reported in the present study were based on the actual projected eroded scar area generated during slurry impingement rather than the total specimen surface area.

### 3.6 SEM surface morphology analysis

Surface morphology of the eroded specimens was examined using scanning electron microscopy (SEM) (JEOL JSM-6360, JEOL Ltd., Japan) operated in secondary electron imaging mode at an accelerating voltage of 15 kV over a magnification range of 100× to 2000 × . Prior to examination, the specimens were ultrasonically cleaned in ethanol and dried to remove loosely adhered debris. SEM examination was employed for qualitative identification of characteristic erosion-induced surface features rather than quantitative phase or compositional analysis. Accordingly, interpretation was based on observable morphological features, including groove formation, ploughing, crater development, cracking, and fragmented debris accumulation, together with the measured erosion trends and established mechanisms reported in the slurry erosion literature.

#### 3.6.1 Brass surface morphology.

**Fig 16:**
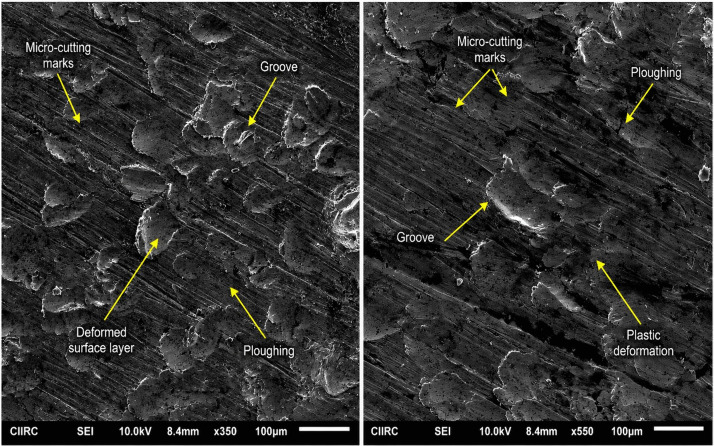
SEM micrographs of brass surfaces after erosion showing groove formation, micro-cutting marks, ploughing, plastic deformation, and localised bright surface regions.

Fig 16(a-b) presents SEM micrographs of eroded brass surfaces obtained under different impingement conditions. At a 30° impingement angle, as shown in Fig 16(a), the surface exhibited elongated erosion-induced grooves, micro-cutting marks, and ploughing features generated by repeated abrasive particle impacts rather than pre-existing material defects. These characteristics are associated with ductile erosion behaviour involving localized plastic deformation and progressive material removal through repeated shearing action. Similar groove formation, ploughing, and surface deformation features have also been reported in tribological and machining investigations of metallic materials subjected to repeated abrasive interactions [56]. The directional nature of the grooves indicates dominant cutting wear under low-angle particle impact conditions.

At a 90° impingement angle, as shown in Fig 16(b), the surface exhibited plastically deformed smeared regions, shallow craters, and irregular protrusions resulting from repeated normal particle impacts. Such morphologies are commonly associated with deformation-dominated erosion mechanisms in ductile materials subjected to near-normal impingement conditions. The observed erosion features demonstrated reasonable agreement with previously reported erosion behaviour of ductile metallic materials under slurry jet erosion conditions [42,44,47].

#### 3.6.2 Cast iron surface morphology.

**Fig 17:**
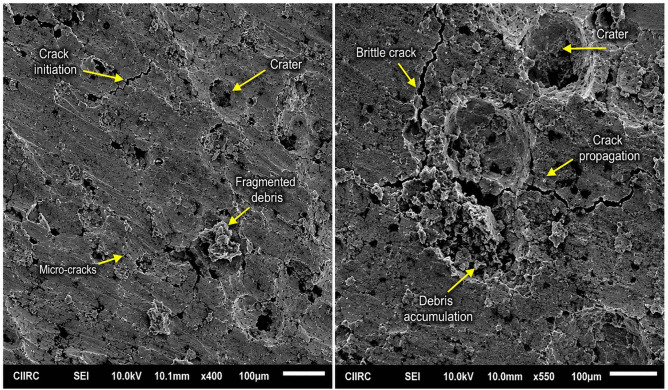
SEM morphology of eroded cast iron surfaces showing (a) crack initiation, micro-crack formation, and localized brittle deformation at 30° impingement angle, and (b) brittle fracture regions, crack propagation, crater formation, and fragmented debris accumulation at 90° impingement angle.

Fig 17(a-b) presents SEM micrographs of eroded cast iron surfaces obtained under different slurry jet erosion conditions. In contrast to brass, cast iron exhibited erosion features associated predominantly with brittle fracture and localized crack propagation. As shown in Fig 17(a), the eroded surface displayed crater formation, fragmented debris accumulation, and shallow fracture regions generated by repeated particle impacts. The limited groove formation observed at lower impingement conditions indicates comparatively lower plastic microstructural deformation behaviour relative to ductile materials.

At near-normal impingement conditions, as shown in Fig 17(b), the surface exhibited extensive micro-cracks, interconnected fracture paths, fragmented erosion debris, and localized brittle surface damage. Repeated particle impacts promoted crack initiation and propagation within the brittle cast iron matrix, leading to localized material detachment and crater formation. In certain regions, compacted debris accumulation around crater boundaries was also observed, indicating localized impact interactions under slurry impingement conditions. These erosion characteristics showed reasonable agreement with previously reported slurry erosion behaviour of brittle metallic materials [44,57–59].

The SEM observations in the present study are interpreted strictly as qualitative morphological evidence. Because EDS and elemental mapping were not performed, no compositional assignment is made for localized bright regions or adhered surface features. Such regions may arise from compacted debris, surface topography, or imaging contrast and are therefore not used to support composition-based conclusions. Similarly, the grooves, craters, and fracture features observed on the eroded surfaces are interpreted as erosion-induced damage generated by repeated abrasive particle impacts rather than pre-existing material defects. The identification of the dominant erosion mechanisms is based on the combined evidence from erosion trends, impingement-angle dependence, material hardness, characteristic post-erosion surface features, and established mechanisms reported in the slurry erosion literature.

### 3.7 Comparison with literature data and test rigs

The performance trends observed using the developed Venturi-based slurry jet erosion test apparatus were compared with previously reported slurry erosion methodologies found in the literature, particularly the slurry erosion methodologies reported by Levy [22], Turenne [23], Hutchings [18]. The Venturi-based configuration simplified the particle introduction mechanism while enabling controlled slurry flow and impingement conditions under laboratory-scale operation. This rig obviates the necessity for intricate gas pressurisation or slurry pumping, which are susceptible to operational irregularities and clogging, in contrast to the pre-mixed and vacuum-ejector approaches. Moreover, the parameters attained-including fluid velocity (6 m/s), particle size (360–655 µm), and slurry concentration (0.1–0.2 wt.%) are either within or beyond the thresholds established by previous test rigs (Tables 1 and 2). The distinct separation between ductile and brittle erosion patterns within the same experimental framework indicates the capability of the rig for comparative laboratory-scale erosion investigations. The results regarding kinetic energy thresholds (brass at about 0.1135 µJ and CI at approximately 1.1572 µJ) align with values documented in previous experimental research [12,52]. The erosion trends related to impingement angle (peaking at 30° for brass and 90^°^ for CI) are in strong agreement with existing erosive behaviour models [44,47].

Although the developed Venturi-based slurry jet erosion test rig demonstrated repeatable laboratory-scale operation and consistent erosion behaviour under controlled laboratory conditions, certain limitations remain. The scope of the present preliminary evaluation was restricted to apparatus development and comparative erosion testing. Accordingly, the test materials were characterised by bulk OES composition, hardness measurements, and qualitative SEM observations, while the erodent was characterised by sieve-based particle-size distribution and SEM morphology. Quantitative metallographic analysis and EDS characterisation of the materials and erodent were not performed. Such analyses would be necessary for studies seeking quantitative correlations among grain structure, phase composition, erodent mineralogy, and erosion rate; however, these correlations were outside the stated objective of the present apparatus-focused investigation. The findings should therefore be interpreted as comparative erosion behaviour under the specified experimental conditions rather than as a comprehensive microstructure- or mineralogy-dependent erosion model. Furthermore, the present configuration operates at a fixed flow velocity of 6 m/s and within a limited slurry concentration range of 0.1–0.2 wt.%, which may not fully represent high-concentration industrial slurry environments. Particle entrainment is achieved through gravity-assisted feeding, which may introduce minor variability in particle delivery at higher flow rates. Future refinements may include extended velocity control ranges, automated particle feeding mechanisms, testing under higher slurry concentrations, and detailed microstructural and compositional characterisation where quantitative relationships among material microstructure, erodent mineralogy, and erosion behaviour are specifically investigated.

## 4 Conclusions

This study presented the design and preliminary experimental evaluation of a Venturi-based slurry jet erosion test apparatus for controlled laboratory-scale erosion investigations. The proposed rig presents a simple and effective alternative to standard setups that depend on gas pressurization or vacuum systems for particle injection using a modified Venturi mechanism. The method enables controlled introduction of dry erosive particles into a high-velocity water stream, permitting comparatively consistent erosion testing across diverse circumstances of particle size, slurry concentration, and impingement angle. Experimental examinations were conducted on two representative metallic materials – brass, a ductile alloy, and CI, a brittle alloy, to assess the versatility and efficacy of the test rig. The research found that brass exhibited maximum material loss at a 30° impingement angle due to dominant micro-cutting and plastic deformation mechanisms, whereas CI exhibited maximum erosion at a 90° impingement angle primarily due to crack initiation, brittle fracture, and material chipping mechanisms. These trends are consistent with classical erosion theories and previously reported literature on ductile and brittle erosion behaviour.

An examination of kinetic energy indicated that the energy threshold necessary to commence erosion in brass was markedly lower (~0.1135 µJ) compared to CI (~1.1572 µJ). This disparity in energy demands underscores the material-specific reaction to erosive wear. The selected particle sizes of 360 µm, 510 µm, and 655 µm generated kinetic energy levels exceeding the identified threshold values, thereby enabling meaningful comparative evaluation under the selected operating conditions. Surface morphology analysis using scanning electron microscopy (SEM) further supported the quantitative erosion results. Brass specimens predominantly exhibited micro-cutting, ploughing grooves, and plastically deformed surface features characteristic of ductile erosion behaviour, whereas CI specimens showed crack formation, brittle fracture regions, impact craters, and fragmented debris accumulation associated with brittle material response. These morphological observations were interpreted qualitatively and were not used to establish quantitative microstructure–erosion or erodent composition–erosion correlations.

The developed Venturi-based slurry jet erosion test rig exhibited repeatable laboratory-scale erosion behaviour and controlled particle entrainment under laboratory-scale operating conditions. The apparatus successfully distinguished the erosion response of ductile and brittle materials across varying impingement angles, slurry concentrations, and particle sizes. The findings suggest that the proposed configuration may serve as a simplified laboratory-scale platform for comparative slurry erosion investigations and future refinement toward broader erosion-testing applications.

## Supporting information

S1 File Representative_supporting_data. (XLSX)
