# Supplementary material for: Development and preliminary experimental evaluation of a venturi-based slurry jet erosion test rig for comparative study of ductile and brittle materials
Source: PLoS One. 2026 Jul 31;21(7):e0355225. doi: 10.1371/journal.pone.0355225 (PMC13426928; doi:10.1371/journal.pone.0355225)
Supplement: Attachment — Submitted filename: Comments.docx [file pone.0355225.s002.docx]

The manuscript has certain errors and suggestions which are in need to be incorporated before the acceptance of the manuscript:

1. The manuscript needs a serious revision by a technical writer. The written language is inconsistent. The irrational use of paraphrasing has deteriorated the readability.
2. In Table 1, the erodent size unit has to be given, and the references need to be properly cited.
3. The objective of the research work should be clearly stated. The author has focused on two diverse things. Design and fabrication of the erosion rig and erosion analysis of materials.
4. Kindly add references in the Cast iron as a brittle material section: “Previous studies [References] show that brittle materials tend to wear out the most at high angles because the impact energy hits them straight on, causing them to break apart and chip.”
5. What do you mean by the term “slurry getting old”?
6. Figure 1 can be presented as a line diagram. The nozzle and stirrer can be illustrated properly.
7. Figure 2 has a bypass valve, and one more valve is also there. What are their functions? What is the nozzle diameter? Kindly add it to the test parameter section. The actual setup picture has to be added along with the schematic diagram.
8. After impingement, the erodent particles are collected in the tank, which is connected to the pump. Does this configuration represent a recirculation-type impingement apparatus? If not, how is it ensured that the used particles re-impinge on the sample?
9. What are the dimensions of erosion test samples? What is the sample preparation procedure? How were the samples extracted? What were the initial conditions of the samples, cast or rolled? Kindly add these details.
10. Specify the type of pump used.
11. Please add an optical microscopy or SEM image of brass and cast iron to understand the microstructure.
12. Use the degree (°) symbol instead of 0 as a superscript.
13. Rephrase the following sentence: “The design follows the basic idea of a Venturi meter, where making the space smaller causes the flow to speed up and the pressure to drop at the narrowest point.”
14. How to vary the wt.% of slurry concentration. Particle induction is solely dependent on the pressure drop at the venturi. How is the flow velocity adjusted?
15. Check the spelling of surface roughness tester. “Tallysurf” or “Talysurf”
16. Indentation resistance is primarily controlled by hardness, not resilience. Check the following sentence and provide a proper citation to corroborate the statement: “More resilient materials generally demonstrate superior resistance to surface deformation and particle indentation, particularly under high-velocity impact scenarios.”
17. Specify the type of Cast iron used. How does an iron matrix become brittle? Check the statement or give the citation to support the statement: “CI has sharp carbides and graphite flakes inside a brittle iron matrix, leading to enhanced resistance to indentation.”
18. Kindly add SEM and EDS analysis of erodent particles. Surface morphology analysis of erodent is necessary because particle morphology influences the erosion behaviour of materials.
19. Check the following sentence and add references to strengthen the statement: “This finding matches what is already known, which shows that ductile materials can wear away with low-energy impacts because of processes like bending and movement of the material.” How does the bending and movement of material help the erosion process?
20. In the following sentence, replace “Zenith” with a suitable word: “As illustrated in Fig 7, weight loss initially escalated and attained a zenith at the 10-minute interval.”
21. Figure 8 Surface roughness of Brass vs. test duration (30 minutes) is missing.
22. Rephrase the following sentence to improve the readability: “An analogous experimental methodology was employed for CI, with assessments performed at a 90^0^-impingement angle, consistent with literature suggesting that maximum attrition in brittle materials transpires under normal impact settings [45].”
23. Check the following sentence: “On the other hand, at a 90^0^- impingement angle, as shown in Fig 13 (c) and Fig 13 (d), the surface has uneven dips, bumps, and areas where the material has flowed.” What material has flowed?
24. Check the following sentence: “At a 30^0^ angle of impact, shown in Figs 14 (a) and 14 (b), the main features are shallow grooves and small cuts, showing that some bending of the material happens at first.” Can a particle impingement cause material to bend? That too with 6 m/s velocity with max. 0.2 wt.% concentration? Explain.
25. Add a subheading “Wear scar analysis” and discuss the scar shape and its relationship with respect to the impingement angle. Photographs depicting the erosion scar will strengthen the manuscript.
26. The erosion scar of a 30° impingement angle looks elliptical in shape, whereas a 90° impingement angle imparts a circular scar. How were the weight loss measurements made? Since the unit mentioned mg/mm^2^, which area was considered? Total area of the sample or the actual eroded area?
27. In Figure 11, at 0.2 wt.% slurry concentration, 510- and 655-micron particle sizes exhibited the same weight loss for 45 to 75° impingement angles; however, at 90° angle, a variation can be seen. Why this disparity? Explain this phenomenon with suitable references.
28. In the eroded surface morphology SEM images, remove the yellow circles. And label the actual erosion mechanisms.
29. The conclusion section should be precise and contain the outcome of the present work.
30. In SEM images, the scale bar is not visible properly. Add a scale bar using ImageJ.
31. Figure 14(a) shows deep grooves and craters. Were they material defects or induced by erodent particles? Figure 14(d) shows white patches. What are they? Kindly add EDS analysis and elemental mapping to better understand the eroded surfaces.
