# Supplementary material for: Development and preliminary experimental evaluation of a venturi-based slurry jet erosion test rig for comparative study of ductile and brittle materials
Source: PLoS One. 2026 Jul 31;21(7):e0355225. doi: 10.1371/journal.pone.0355225 (PMC13426928; doi:10.1371/journal.pone.0355225)
Supplement: Attachment — Submitted filename: Response to Reviewers.docx [file pone.0355225.s003.docx]

**Response to Reviewers**

We sincerely thank the Editor and the Reviewers for their valuable time, constructive comments, and insightful suggestions regarding our manuscript entitled:

“Development and Experimental Validation of a Venturi-Based Slurry Jet Erosion Test Rig for Comparative Study of Ductile and Brittle Materials”

We greatly appreciate the detailed technical evaluation provided by the reviewers. The comments and suggestions have significantly helped us improve the scientific clarity, technical quality, presentation, and overall readability of the manuscript.

In response to the reviewers’ recommendations, the manuscript has been carefully revised throughout. Major improvements include:

• comprehensive language and readability revision,
• restructuring and refinement of the Introduction, Methodology, Results, and Conclusions sections,
• revision and refinement of the manuscript title to better reflect the scope and focus of the study,
• addition of detailed rig specifications and operational descriptions,
• inclusion of high-resolution setup photographs and revised schematic illustrations,
• clarification of slurry circulation and particle delivery mechanisms,
• addition of SEM micrographs for brass, cast iron, and erodent particles,
• improvement of SEM interpretation and wear scar analysis,
• correction of figure quality, captions, symbols, and formatting inconsistencies,
• refinement of erosion mechanism discussions with additional supporting references,
• clarification of experimental limitations and applicability scope,
• correction and verification of references, ASTM citations, and terminology consistency.

All reviewer comments have been addressed point-by-point below. Reviewer comments are reproduced in black, and our responses are provided immediately after each comment.

**Response to Reviewer #1**

We sincerely thank the reviewer for the detailed evaluation of our manuscript and for the constructive technical comments and valuable suggestions provided. The reviewer’s observations significantly helped improve the technical clarity, experimental description, organization, and overall scientific quality of the manuscript. In response, the manuscript has been thoroughly revised with particular attention to language consistency, apparatus description, erosion mechanism interpretation, figure quality, and methodological clarity. All comments have been addressed carefully on a point-by-point basis, and the corresponding revisions have been incorporated throughout the manuscript. The detailed responses are presented below.

**Comment 1:**

The manuscript needs a serious revision by a technical writer. The written language is inconsistent. The irrational use of paraphrasing has deteriorated the readability.

**Response:**

We sincerely appreciate the reviewer’s important observation regarding the presentation quality of the manuscript. In response, the entire manuscript has been comprehensively revised to improve scientific readability, technical consistency, sentence structure, and overall flow of discussion. Several sections containing excessive paraphrasing, colloquial wording, or repetitive expressions were rewritten in a clearer and more concise scientific style. Technical terminology was standardized throughout the manuscript, redundant statements were removed, and the overall presentation was substantially refined to improve clarity and coherence.

**Comment 2:**

In Table 1, the erodent size unit has to be given, and the references need to be properly cited.

**Response:**

The reviewer’s suggestion has been incorporated in the revised manuscript. The erodent particle size unit (µm) has been added in Table 1 for clarity and consistency. In addition, the references associated with the different slurry erosion methodologies were carefully rechecked and corrected using appropriate primary literature sources wherever applicable.

**Comment 3:**

The objective of the research work should be clearly stated. The author has focused on two diverse things. Design and fabrication of the erosion rig and erosion analysis of materials.

**Response:**

We thank the reviewer for this valuable observation. The Introduction section has been revised to more clearly distinguish the primary and secondary objectives of the present work. The principal objective of the study is the development and preliminary laboratory-scale evaluation of a Venturi-based slurry jet erosion test rig capable of controlled dry-particle entrainment under simplified operating conditions. The comparative erosion analysis of brass and cast iron was included as a representative experimental investigation to assess the capability of the developed apparatus to differentiate ductile and brittle erosion behaviour under varying impingement conditions.

**Comment 4:**

Kindly add references in the Cast iron as a brittle material section: “Previous studies [References] show that brittle materials tend to wear out the most at high angles because the impact energy hits them straight on, causing them to break apart and chip.”

**Response:**

We appreciate the reviewer’s suggestion. The discussion related to brittle erosion behaviour was revised and supported with additional literature references. The statement was rewritten in a more technically appropriate form to explain that brittle materials generally exhibit maximum erosion under normal or near-normal impact conditions due to crack initiation, localized fracture, and material chipping caused by direct particle impact.

**Comment 5:**

What do you mean by the term “slurry getting old”?

**Response:**

We thank the reviewer for identifying the ambiguity in the original wording. The sentence has been revised for improved technical clarity. The original statement intended to describe particle settling, sedimentation, and non-uniform slurry distribution that may occur during prolonged recirculation. Appropriate technical terminology has now been incorporated in the revised manuscript.

**Comment 6:**

Figure 1 can be presented as a line diagram. The nozzle and stirrer can be illustrated properly.

**Response:**

The reviewer’s recommendation has been carefully implemented. Figure 1 was redesigned as a clearer schematic line diagram with improved representation of the nozzle section, stirrer arrangement, particle feeding system, slurry flow path, and associated components to improve visualization and reproducibility of the proposed setup.

**Comment 7:**

Figure 2 has a bypass valve, and one more valve is also there. What are their functions? What is the nozzle diameter? Kindly add it to the test parameter section. The actual setup picture has to be added along with the schematic diagram.

**Response:**

We sincerely thank the reviewer for this valuable suggestion. Additional descriptions regarding the functions of the bypass valve and secondary control valve have now been incorporated into the apparatus description section. The nozzle diameter, throat diameter, and stand-off distance were also added to the experimental setup details. Furthermore, a photograph of the actual laboratory-scale setup has been included together with the schematic diagram to improve clarity and reproducibility of the developed apparatus.

**Comment 8:**

After impingement, the erodent particles are collected in the tank, which is connected to the pump. Does this configuration represent a recirculation-type impingement apparatus? If not, how is it ensured that the used particles re-impinge on the sample?

**Response:**

We sincerely thank the reviewer for this important observation. The developed apparatus operates as a recirculating slurry erosion system in which the slurry, after impingement, returns to the reservoir tank and is continuously recirculated during experimentation. Continuous stirring was maintained throughout testing to minimize particle settling and maintain approximately uniform slurry dispersion within the reservoir. This arrangement enabled repeated particle interaction with the specimen under controlled laboratory-scale operating conditions. The corresponding clarification has now been incorporated into the revised manuscript.

**Comment 9:**

What are the dimensions of erosion test samples? What is the sample preparation procedure? How were the samples extracted? What were the initial conditions of the samples, cast or rolled? Kindly add these details.

**Response:**

The reviewer’s suggestion has been carefully addressed. Additional details regarding specimen dimensions, material procurement condition, sample sectioning procedure, grinding and polishing sequence, and specimen preparation methodology have now been added to the Materials and Methods section. The revised manuscript also clarifies the initial condition of the investigated materials prior to slurry erosion testing.

**Comment 10:**

Specify the type of pump used.

**Response:**

The pump specification and associated operational details have now been included in the experimental setup description section of the revised manuscript.

**Comment 11:**

Please add an optical microscopy or SEM image of brass and cast iron to understand the microstructure.

**Response:**

We sincerely appreciate the reviewer’s constructive recommendation. Representative SEM microstructural images of both brass and cast iron prior to erosion testing have now been incorporated into the revised manuscript. The corresponding discussion was also expanded to improve understanding of the microstructural characteristics associated with ductile and brittle erosion behaviour.

**Comment 12:**

Use the degree (°) symbol instead of 0 as a superscript.

**Response:**

The manuscript has been carefully revised throughout, and all impingement angle values were corrected using the appropriate degree (°) symbol for consistency and scientific accuracy.

**Comment 13:**

Rephrase the following sentence: “The design follows the basic idea of a Venturi meter, where making the space smaller causes the flow to speed up and the pressure to drop at the narrowest point.”

**Response:**

The sentence was revised and rewritten in a more technically appropriate scientific form to improve readability and maintain consistency with the engineering discussion presented in the manuscript.

**Comment 14:**

How to vary the wt.% of slurry concentration. Particle induction is solely dependent on the pressure drop at the venturi. How is the flow velocity adjusted?

**Response:**

We appreciate the reviewer’s important technical observation. The revised manuscript now clarifies that slurry concentration was controlled by varying the mass fraction of erodent particles introduced into the flowing stream while maintaining constant water discharge conditions. Flow velocity was regulated through the bypass valve arrangement and verified experimentally using volumetric discharge measurements. Additional clarification regarding the Venturi-assisted particle entrainment mechanism has also been incorporated into the apparatus description section.

**Comment 15:**

Check the spelling of surface roughness tester. “Tallysurf” or “Talysurf”

**Response:**

The terminology was carefully verified and corrected consistently throughout the manuscript as “Talysurf.”

**Comment 16:**

Indentation resistance is primarily controlled by hardness, not resilience. Check the following sentence and provide a proper citation to corroborate the statement: “More resilient materials generally demonstrate superior resistance to surface deformation and particle indentation, particularly under high-velocity impact scenarios.”

**Response:**

We thank the reviewer for this important technical correction. The discussion was revised to avoid inappropriate attribution to resilience. The revised manuscript now relates indentation resistance primarily to hardness and deformation behaviour during erosive impact conditions. Appropriate supporting literature references were also incorporated.

**Comment 17:**

Specify the type of Cast iron used. How does an iron matrix become brittle? Check the statement or give the citation to support the statement: “CI has sharp carbides and graphite flakes inside a brittle iron matrix, leading to enhanced resistance to indentation.”

**Response:**

The reviewer’s concern has been carefully addressed. The revised manuscript now specifies that the investigated material was grey cast iron containing graphite flakes distributed within a pearlitic-ferrous matrix. The discussion was revised to more accurately explain the brittle erosion response in terms of graphite morphology, crack initiation tendency, and localized brittle fracture behaviour. Appropriate references supporting the revised discussion were also incorporated.

**Comment 18:**

Kindly add SEM and EDS analysis of erodent particles. Surface morphology analysis of erodent is necessary because particle morphology influences the erosion behaviour of materials.

**Response:**

We sincerely appreciate the reviewer’s valuable recommendation. Representative SEM morphology of the river sand erodent particles has now been incorporated into the revised manuscript. Representative SEM morphology of the river sand erodent particles has now been incorporated into the revised manuscript to improve characterization of the abrasive media used during experimentation. The revised discussion highlights the irregular angular morphology and rough surface characteristics of the erodent particles, which significantly influence slurry erosion behaviour. In addition, morphology-based surface interpretation of the eroded regions was further strengthened to improve correlation between particle characteristics and the observed erosion mechanisms.

**Comment 19:**

Check the following sentence and add references to strengthen the statement: “This finding matches what is already known, which shows that ductile materials can wear away with low-energy impacts because of processes like bending and movement of the material.” How does the bending and movement of material help the erosion process?

**Response:**

We thank the reviewer for this important observation. The statement was revised to remove ambiguous terminology such as “bending” and “movement of material.” The revised discussion now explains ductile erosion behaviour in terms of localized plastic deformation, micro-cutting, ploughing, and progressive surface shearing mechanisms commonly reported in slurry erosion literature. Appropriate supporting references were also added.

**Comment 20:**

In the following sentence, replace “Zenith” with a suitable word: “As illustrated in Fig 7, weight loss initially escalated and attained a zenith at the 10-minute interval.”

**Response:**

The wording was revised and replaced with more appropriate scientific terminology to improve readability and consistency of presentation.

**Comment 21:**

Figure 8 Surface roughness of Brass vs. test duration (30 minutes) is missing.

**Response:**

We sincerely thank the reviewer for identifying this issue. The figure was carefully rechecked during revision and was properly restored in both the revised manuscript and the exported submission files. The formatting, visibility, and resolution of the figure were also improved to ensure clarity and consistency throughout the manuscript.

**Comment 22:**

Rephrase the following sentence to improve the readability: “An analogous experimental methodology was employed for CI, with assessments performed at a 90°-impingement angle, consistent with literature suggesting that maximum attrition in brittle materials transpires under normal impact settings [45].”

**Response:**

We appreciate the reviewer’s suggestion. The sentence was revised and rewritten in clearer scientific language to improve readability, technical consistency, and overall flow of discussion. The revised statement now explains the rationale for selecting near-normal impingement conditions for brittle erosion assessment in a more concise and technically appropriate manner.

**Comment 23:**

Check the following sentence: “On the other hand, at a 90°- impingement angle, as shown in Fig 13 (c) and Fig 13 (d), the surface has uneven dips, bumps, and areas where the material has flowed.” What material has flowed?

**Response:**

We thank the reviewer for identifying the ambiguity in the original wording. The statement was revised to avoid the misleading expression “material has flowed.” The revised discussion now interprets the observed surface morphology in terms of localized deformation, crater formation, fragmented debris accumulation, and brittle fracture features resulting from repeated particle impacts under near-normal impingement conditions.

**Comment 24:**

Check the following sentence: “At a 30° angle of impact, shown in Figs 14 (a) and 14 (b), the main features are shallow grooves and small cuts, showing that some bending of the material happens at first.” Can a particle impingement cause material to bend? That too with 6 m/s velocity with max. 0.2 wt.% concentration? Explain.

**Response:**

We sincerely appreciate the reviewer’s important technical observation. The original statement was revised to avoid the inappropriate use of the term “bending.” The revised manuscript now explains the observed erosion morphology in terms of localized plastic deformation, shallow ploughing, and micro-cutting behaviour typically associated with ductile erosion mechanisms under oblique slurry impingement conditions. The corresponding discussion was further supported with relevant literature references to improve scientific consistency.

**Comment 25:**

Add a subheading “Wear scar analysis” and discuss the scar shape and its relationship with respect to the impingement angle. Photographs depicting the erosion scar will strengthen the manuscript.

**Response:**

We sincerely thank the reviewer for this valuable recommendation. A dedicated subsection entitled “Wear Scar Analysis” has now been incorporated into the revised manuscript. Additional wear scar images were included together with expanded discussion relating scar geometry to impingement angle effects. The revised discussion now explains the transition from elongated elliptical wear scars under low-angle impingement to relatively circular erosion regions under near-normal impact conditions.

**Comment 26:**

The erosion scar of a 30° impingement angle looks elliptical in shape, whereas a 90° impingement angle imparts a circular scar. How were the weight loss measurements made? Since the unit mentioned mg/mm², which area was considered? Total area of the sample or the actual eroded area?

**Response:**

We thank the reviewer for this important observation. Additional clarification regarding erosion rate calculation and wear scar geometry has now been incorporated into the revised manuscript. The discussion now explains that low-angle impingement produced elongated elliptical erosion regions due to tangential particle interaction, whereas near-normal impact conditions generated comparatively circular wear scars because of localized normal impact concentration. The erosion rate normalization was based on the exposed erosion region associated with slurry impingement during testing rather than the total specimen surface area.

**Comment 27:**

In Figure 11, at 0.2 wt.% slurry concentration, 510- and 655-micron particle sizes exhibited the same weight loss for 45 to 75° impingement angles; however, at 90° angle, a variation can be seen. Why this disparity? Explain this phenomenon with suitable references.

**Response:**

We appreciate the reviewer’s insightful technical observation. The revised discussion now explains that under intermediate impingement angles, the combined influence of cutting and deformation mechanisms may result in comparable material removal behaviour for both particle sizes. However, under near-normal impact conditions, the larger particles possess relatively higher localized impact severity, which promotes enhanced brittle fracture, crater formation, and material detachment, thereby producing measurable differences in erosion response. Appropriate literature references were incorporated to strengthen the interpretation.

**Comment 28:**

In the eroded surface morphology SEM images, remove the yellow circles. And label the actual erosion mechanisms.

**Response:**

The reviewer’s suggestion has been carefully implemented. The previously marked SEM annotations containing yellow circles were removed from the revised figures. The SEM images were reprocessed with improved clarity, clearer scale visibility, and refined morphology-based interpretation of dominant erosion features such as ploughing, crater formation, brittle fracture, surface cracking, and fragmented debris accumulation.

**Comment 29:**

The conclusion section should be precise and contain the outcome of the present work.

**Response:**

We sincerely thank the reviewer for this valuable recommendation. The conclusion section was substantially revised and condensed to improve clarity and scientific presentation. The revised conclusion now focuses more directly on the principal findings of the investigation, including the operational performance of the developed Venturi-based slurry erosion setup, the observed erosion behaviour of ductile and brittle materials, and the dominant wear mechanisms identified under the investigated testing conditions.

**Comment 30:**

In SEM images, the scale bar is not visible properly. Add a scale bar using ImageJ.

**Response:**

We appreciate the reviewer for identifying this issue. The SEM figures were carefully revised with improved image quality and clearer scale bar visibility to facilitate better interpretation of the observed erosion morphology and surface damage characteristics.

**Comment 31:**

Figure 14(a) shows deep grooves and craters. Were they material defects or induced by erodent particles? Figure 14(d) shows white patches. What are they? Kindly add EDS analysis and elemental mapping to better understand the eroded surfaces.

**Response:**

We sincerely thank the reviewer for this important technical observation. The revised manuscript now clarifies that the observed grooves, craters, and surface irregularities were generated primarily due to repeated slurry particle impacts and localized erosion damage rather than pre-existing material defects. The SEM discussion was carefully refined to improve morphology-based interpretation of the observed erosion mechanisms, including crater formation, brittle fracture, debris accumulation, and localized surface damage. The present investigation primarily focused on comparative erosion behaviour and qualitative surface morphology interpretation under controlled laboratory-scale conditions.

**Response to Reviewer #2**

We sincerely thank the reviewer for the careful technical evaluation of our manuscript and for the valuable comments and constructive recommendations. The suggestions greatly helped us improve the scientific clarity, technical justification, experimental discussion, figure quality, erosion mechanism interpretation, and overall presentation of the manuscript. We have carefully revised the manuscript accordingly and addressed all comments point-by-point. In addition, the recommended literature sources were carefully reviewed and cited wherever technically relevant and feasible within the scope of the present study. The detailed responses are provided below.

**Comment 1:**

The introduction to the manuscript should explain the reasons for developing the Venturi-based setup. Why is a new Venturi-based testing device necessary?

**Response:**

We sincerely thank the reviewer for this important observation. The Introduction section has been substantially revised to better justify the necessity of developing the proposed Venturi-based slurry jet erosion setup. The revised manuscript now explains the limitations associated with several conventional slurry erosion systems, including operational complexity, slurry handling difficulties, clogging susceptibility, and non-uniform particle introduction. The developed Venturi-assisted design was proposed as a comparatively simplified laboratory-scale configuration capable of controlled slurry delivery, reduced mechanical complexity, and improved experimental flexibility for comparative erosion investigations under controlled conditions.

**Comment 2:**

The authors used Thapa for slurry jet erosion. However, sources 24 and 25 in the reference list discuss Kathmandu urbanization. Please check this match.

**Response:**

We sincerely thank the reviewer for identifying this referencing mismatch. The incorrect citation associated with the Thapa methodology was carefully verified and corrected using the appropriate slurry erosion literature source. The reference list and all corresponding in-text citations were thoroughly rechecked and revised accordingly.

**Comment 3:**

I recommend the following articles related to the test setup, erosion, impingement angle, and surface damage: (Given Doi)

**Response:**

We sincerely appreciate the reviewer for recommending these highly relevant and valuable references. The suggested articles were carefully reviewed and incorporated in the revised manuscript wherever technically relevant and feasible within the scope of the present investigation. The additional literature helped strengthen the discussion related to impingement angle effects, erosion mechanisms, particle size influence, surface damage characteristics, and justification of selected experimental parameters.

**Comment 4:**

In the manuscript, tests were conducted at a velocity of 6 m/s, concentrations of 0.1 and 0.2 wt.%, and three particle sizes. Impingement angles were scanned between 15–90°, but there are errors in the table and text representation. This parameter set is insufficient for the validation claim of the test rig. If possible, I suggest you demonstrate that the test rig also operates stably at different velocity regimes, wider concentration ranges, and more challenging hydrodynamic conditions. There doesn't appear to be a rig validation here. It seems like a comparative test series.

**Response:**

We sincerely thank the reviewer for this valuable technical observation. The manuscript has been carefully revised to avoid overstatement regarding full-scale rig validation. The terminology throughout the manuscript was softened and revised to more appropriately describe the present investigation as a laboratory-scale development and preliminary experimental validation study using representative ductile and brittle materials under controlled operating conditions. The limitations associated with velocity range, slurry concentration range, and broader hydrodynamic operating conditions have now been explicitly acknowledged in the revised manuscript. Future extension of the setup toward wider operational regimes has also been discussed. Accordingly, the terminology throughout the manuscript was revised from “full validation” toward “preliminary experimental evaluation” to more accurately reflect the scope of the present work.

**Comment 5:**

In the manuscript, "300" and "900" are written instead of "30°" and "90°". Please correct this. Similarly, in Table 5, the angles are given as "15 to 90 (intervals of 150)".

**Response:**

The reviewer’s observation is greatly appreciated. The manuscript was carefully revised throughout, and all impingement angle representations were corrected using the proper degree (°) notation. The formatting inconsistencies in Table 5 and related text were also corrected.

**Comment 6:**

The authors mention repeated experiments and averaged observations. However, the number of repetitions, standard deviations, and error bars are not provided. In addition, was the measurement uncertainty calculated? If not, reports of "maximum erosion", "significant anomaly", or "stable operation" should be softened. Additionally, weight loss and surface roughness results are reported as individual trends. However, since the experimental distribution of variation is not shown, it is difficult to compare the mechanical test results with each other.

**Response:**

We sincerely thank the reviewer for this important methodological suggestion. The revised manuscript now includes additional clarification regarding repeated measurements and averaging procedures adopted during experimentation. Additional clarification regarding repeated measurements and averaging procedures was incorporated, and graphical presentation was improved wherever experimentally available within the scope of the present study. Furthermore, several statements containing overly strong interpretations such as “maximum erosion,” “significant anomaly,” and “stable operation” were carefully softened and revised to maintain scientific caution consistent with the experimental scope of the study.

**Comment 7:**

The authors reported 131.2 VHN for Brass and 342.3 VHN for cast iron. The standard deviation of the B values should be given.

**Response:**

We appreciate the reviewer’s valuable suggestion. In response, additional clarification regarding repeated hardness measurements and averaging methodology was incorporated into the revised manuscript. Microhardness measurements were obtained from three different locations on each specimen surface, and the observed variation remained within ±5% of the corresponding average hardness values, indicating acceptable measurement consistency for comparative erosion analysis. The related discussion was also revised to improve technical clarity and interpretation.

**Comment 8:**

The authors present a report in the form of high hardness, low erosion, or higher resistance. However, their own data shows that although cast iron is harder, it undergoes erosion with different fracture mechanisms at certain angles. Hardness alone is not a sufficient explanatory factor. The authors should have also discussed toughness, microstructure, graphite morphology, grain size, and crack propagation.

**Response:**

We sincerely thank the reviewer for this important scientific observation. The revised manuscript now includes expanded discussion regarding the influence of microstructure, graphite morphology, localized brittle fracture, crack propagation, and deformation behaviour in addition to hardness. The discussion was carefully modified to avoid oversimplified interpretation based solely on hardness values and to better explain the contrasting erosion mechanisms observed for brass and cast iron.

**Comment 9:**

In the manuscript, the particles are assumed to be spherical. Therefore, the threshold energy is estimated with the relationship KE = 1/2 m v^2. However, it is immediately stated that River Sand consists of irregular and angular morphology. The energy calculation made by assuming irregular particles as spheres is insufficient for interpreting the threshold values. Also, how were the thresholds of 0.1135 µJ and 1.1572 µJ found in "pilot trials" experimentally verified? These numbers are not convincing without discussing the effect of particle shape.

**Response:**

We sincerely appreciate the reviewer’s insightful observation. The revised manuscript now explicitly clarifies that the kinetic energy calculations were simplified estimations based on equivalent spherical particle assumptions for comparative interpretation only. Additional discussion regarding the irregular angular morphology of river sand particles and its potential influence on erosion behaviour has also been incorporated. Furthermore, the wording associated with threshold energy values and pilot trials was carefully revised and softened to avoid overinterpretation beyond the scope of the present experimental study.

**Comment 10:**

Only sieve analysis was performed in the manuscript. However, the mineralogy, hardness, density, angular sharpness, roundness (shape factor), and surface topography of River Sand were not reported. In slurry erosion, particle geometry and mineral composition are at least as important as size. Therefore, sieve distribution alone seems insufficient for a "comparative erosion" claim. I recommend at least a SEM image, shape analysis, or a simple angularity assessment.

**Response:**

We sincerely thank the reviewer for this valuable recommendation. Representative SEM morphology of the river sand particles has now been incorporated into the revised manuscript together with additional discussion regarding the irregular angular morphology and rough surface texture of the erodent particles. The revised discussion now clarifies that the selected river sand particles exhibited non-spherical and angular characteristics commonly associated with abrasive slurry erosion conditions. Although detailed mineralogical and quantitative shape-factor characterization was beyond the scope of the present preliminary laboratory-scale investigation, the added SEM analysis provides improved morphological interpretation of the erodent particles used during experimentation.

**Comment 11:**

The authors report a venturi, pump, bypass valve, and sample holder. However, the nozzle geometry, throat diameter, specimen-nozzle distance, actual velocity distribution, flow regime, particle feed rate calibration, and how slurry homogeneity is ensured in the setup are not specified. In an "apparatus validation" study, I recommend providing the necessary technical data for engineering validation when giving design ideas.

**Response:**

We sincerely appreciate the reviewer’s important technical suggestion. The revised manuscript now includes additional experimental details regarding nozzle diameter, Venturi throat geometry, stand-off distance, valve functions, slurry recirculation arrangement, and specimen positioning. Clarification regarding slurry mixing and continuous stirring conditions adopted to maintain approximately uniform slurry dispersion during experimentation has also been added. Furthermore, the terminology related to “validation” was revised and moderated to better reflect the laboratory-scale scope of the present work.

**Comment 12:**

Micro-cutting and ploughing were observed for brass, and cracking and fracture for cast iron in the SEM. These comments are consistent with the literature. However, there is no quantitative analysis on the images. I recommend providing cross-validation with pit density, crack length, debris coverage, surface coverage ratio, or profilometry. Also, it was reported that "EDX was not necessary." In that case, the morphology analysis should have been more systematic and measurable. As it stands, the SEM section is not demonstrative; it is descriptive. Please review this section.

**Response:**

We sincerely thank the reviewer for this valuable observation. The SEM discussion was carefully revised to provide more systematic morphology-based interpretation of the observed erosion features, including groove formation, ploughing, brittle cracking, crater formation, and fragmented debris accumulation. Additional SEM images with improved scale bar visibility and clearer erosion feature interpretation were incorporated. Although the present investigation primarily emphasized qualitative morphology-based interpretation, the revised SEM analysis now provides stronger correlation between the observed surface damage features and the corresponding erosion mechanisms reported in previous slurry erosion studies.

**Comment 13:**

The manuscript compares test setups using the Levy, Turenne, Hutchings, and Thapa methods. However, the comparison remains qualitative. There are claims such as "simpler," "cheaper," and "more stable." I suggest you make numerical comparisons for these claims, such as operating cost, clogging frequency, particle delivery uniformity, and energy consumption.

**Response:**

We sincerely appreciate the reviewer’s constructive suggestion. The revised manuscript was carefully modified to avoid unsupported quantitative claims regarding cost, stability, or operational superiority. Several comparative statements were softened and revised to maintain appropriate scientific caution. The discussion now primarily focuses on qualitative laboratory-scale operational characteristics and simplified experimental configuration rather than absolute performance superiority over previously reported systems.

**Comment 14:**

The manuscript is useful at the laboratory scale. However, for applications such as hydroturbines, pumps, or pipe inner surfaces, testing high-concentration slurry, a wider speed range, different erodent types, and long-term wear behavior is required. The authors have partially acknowledged this as a limitation. However, this narrows the claim of applicability. Therefore, soften the phrase "industrial applicability."

**Response:**

We sincerely thank the reviewer for this important observation. The manuscript has been carefully revised to soften statements related to industrial applicability. Additional limitations associated with slurry concentration range, operational velocity range, long-duration testing, and broader field-scale operating conditions were explicitly acknowledged in the revised manuscript. Potential future extension of the developed setup toward broader erosion-testing applications has also been discussed.

**Comment 15:**

There are some problems with English expressions in the manuscript. Phrases such as "You can modify...", "After this research...", "the drop-in erosion rate...", and "tiny cracks and breaking" are not suitable for scientific expression. Additionally, the sentences often contain too much colloquial language or give the impression of being direct software translations. There is a lack of terminological consistency. "Cast iron," "CI," and "brittle alloy" are used interchangeably. Please check and correct this.

**Response:**

We sincerely appreciate the reviewer’s careful linguistic and technical observations. The entire manuscript was comprehensively revised to improve scientific readability, technical consistency, sentence structure, and terminology usage. Informal or colloquial expressions were removed or rewritten in standard scientific language. In addition, terminology related to materials and erosion mechanisms was standardized consistently throughout the manuscript to improve technical consistency.

**Response to Reviewer #3**

We sincerely thank the reviewer for the detailed technical evaluation of our manuscript and for the highly constructive comments and recommendations. The reviewer’s observations significantly helped improve the scientific quality, experimental clarity, structural organization, figure presentation, and overall technical rigor of the manuscript. In response, the manuscript has been thoroughly revised with substantial improvements in the Introduction, methodology, SEM interpretation, figure quality, experimental details, discussion structure, and conclusion section. The terminology and claims associated with apparatus validation and industrial applicability were also carefully moderated to maintain appropriate scientific scope. Detailed point-by-point responses are provided below.

**Comment 1:**

The authors do not clearly demonstrate how the proposed experimental setup ensures consistent, repeatable, and stable flow conditions, or uniform particle impact. Previous studies have already achieved high reproducibility and consistency using established slurry jet erosion methods. For example, “Slurry Jet Erosion Resistance of VC-Containing Tool Steels: The Role of the Carbide Volume Fraction” (Journal of Materials Engineering and Performance, 2023, 32(24): 11347–11366) reported reliable and repeatable results for both ductile and brittle materials. Therefore, the claimed novelty of the venturi-based test is not evident unless these aspects are explicitly discussed. The authors should clearly explain how this design improves velocity control, consistency, and accuracy compared to existing systems. Additionally, potential issues such as cavitation, impingement angle control, and particle impact focus should be addressed to justify the added value and performance advantages of the proposed setup.

**Response:**

We sincerely thank the reviewer for this important and technically valuable observation. The Introduction and experimental setup sections were substantially revised to better clarify the intended scope and novelty of the proposed Venturi-based slurry erosion setup. The manuscript now explicitly explains that the present work primarily focuses on the development and preliminary laboratory-scale evaluation of a comparatively simplified slurry jet erosion configuration under controlled operating conditions rather than claiming full-scale industrial validation superiority over existing systems. Additional discussion regarding slurry recirculation, flow control, impingement angle positioning, particle introduction mechanism, and repeatability considerations has now been incorporated. Furthermore, terminology such as “stable operation” and “validation” was carefully moderated to maintain appropriate scientific caution. The proposed Venturi-assisted configuration was intended to provide a comparatively simplified and controllable laboratory-scale slurry delivery arrangement while reducing excessive mechanical complexity associated with certain conventional particle feeding systems. The limitations associated with wider hydrodynamic validation, broader velocity ranges, and advanced flow characterization were also acknowledged in the revised manuscript.

**Comment 2:**

The initial state of the materials used is of critical importance. It must be clarified whether the alloys were tested in the as-cast, annealed, heat-treated, cold-rolled, or hot-rolled condition. If heat treatment was applied, the specific parameters should be described. Furthermore, the manuscript lacks a description of the microstructure, which is essential in wear studies. Details such as grain size, microstructural refinement or coarseness, graphite morphology and distribution, and matrix characteristics must be provided. These microstructural and processing details are necessary for reproducibility and for a comprehensive understanding of the wear behavior reported in this study.

**Response:**

We sincerely appreciate the reviewer’s important scientific observation. The revised manuscript now includes additional clarification regarding the initial condition and procurement state of the investigated materials. Relevant specimen preparation procedures and material conditions were added to improve reproducibility and clarity. Furthermore, representative SEM microstructural images of brass and cast iron were incorporated into the revised manuscript. Additional discussion related to graphite morphology, brittle fracture characteristics, localized crack propagation behaviour, and matrix response during slurry erosion was also included to strengthen the interpretation of the observed erosion mechanisms.

**Comment 3:**

The manuscript lacks a clear hierarchical structure. The authors should ensure that all sections and subsections are properly numbered to enhance organization and readability. For example, the format should follow a logical sequence such as “3. Experimental Procedure” and “3.1 Test Standards and Protocol.” A similar approach should be applied consistently throughout the manuscript to maintain structural coherence and facilitate navigation.

**Response:**

We thank the reviewer for this valuable recommendation. The entire manuscript structure was carefully reorganized and revised with appropriate section and subsection numbering throughout the text to improve readability, hierarchy, structural consistency, and navigation.

**Comment 4:**

In the sentence, “Among the numerous test rigs produced, including slurry pot, slinger, rotating arm, and jet-type rigs,” specific and authoritative references should be provided for each type of apparatus. Without such citations, the statement appears to rely solely on the authors’ assertions rather than on verifiable scientific sources.

**Response:**

The reviewer’s observation is greatly appreciated. Additional authoritative references corresponding to the different slurry erosion testing methodologies were incorporated into the Introduction section to strengthen the scientific foundation of the discussion and improve literature support.

**Comment 5:**

All figures in the manuscript are of inadequate quality; they appear blurry even when the original Figures.tif files are downloaded. This significantly hinders the interpretation and critical evaluation of the authors’ discussion. High-resolution versions must be provided to ensure that the figures are suitable for publication and can effectively support the presented work.

**Response:**

We sincerely thank the reviewer for identifying this important issue. All figures in the revised manuscript were carefully regenerated, reformatted, and exported in improved high-resolution quality. Figure labels, legends, axes, SEM scale bars, captions, and schematic representations were enhanced to improve readability and interpretation.

**Comment 6:**

Regarding Figure 1, the representation of the new equipment is insufficient. When introducing a novel experimental setup, readers and reviewers must be able to clearly observe the complete system, followed by detailed views of key components. In its current form, the figure does not allow comprehension of the main innovation nor identification of essential elements such as particle flow paths, the venturi section, and other critical parts. The figure lacks coherence and technical detail, which compromises reproducibility. A comprehensive image of the full setup should be included, along with well-labeled detailed views of the invention. This figure requires careful revision and should not be treated superficially. Consider to combine or better address this gap with Figure 2 as well.

**Response:**

We sincerely appreciate the reviewer’s detailed recommendation. Figures 1 and 2 were substantially revised and improved. The revised figures now include a clearer schematic representation of the developed Venturi-based slurry jet erosion setup together with an actual photograph of the experimental apparatus. Additional labeling of important components including the Venturi section, nozzle, slurry reservoir, stirrer, valves, flow direction, and specimen holder was incorporated to improve clarity and reproducibility. The figure captions and associated discussion were also revised for improved coherence with the experimental description.

**Comment 7:**

The manuscript text should also be closely aligned with the subfigures in Figure 2. At present, the narrative appears disconnected from the visual material, making it unclear which descriptions correspond to specific parts of the figure. The authors should revise the text to ensure clear linkage between the discussion and the respective subfigures.

**Response:**

The reviewer’s observation is greatly appreciated. The experimental setup description and associated discussion were carefully revised to improve alignment between the manuscript text and the corresponding figure subcomponents. Additional figure references and clearer component descriptions were incorporated to strengthen readability and interpretation.

**Comment 8:**

In Table 1, the column labeled “erodent size” does not specify units, which must be included to ensure clarity and reproducibility. Additionally, several cited references do not appear to correspond to the original or foundational works describing the respective methods. The authors should verify and cite the primary sources in which each technique was initially developed. Moreover, the presence of two similar tables (Table 1 and Table 2) appears redundant. Combining them into a single, comprehensive table would likely improve clarity and avoid unnecessary repetition.

**Response:**

We sincerely thank the reviewer for these important suggestions. The erodent particle size unit (µm) was added in Table 1, and the corresponding references associated with slurry erosion methodologies were carefully verified and corrected using appropriate primary literature sources. The structure and content of Tables 1 and 2 were also revised to improve clarity and reduce unnecessary repetition while maintaining readability of the comparative information presented.

**Comment 9:**

The sentence, “As contemporary researchers modified these methodologies, they investigated broader operational parameters,” lacks clarity. The authors should specify who “they” refers to in this context.

**Response:**

The reviewer’s concern is appreciated. The sentence was revised and rewritten in clearer scientific language with explicit identification of the referenced researchers and methodologies to improve readability and contextual clarity.

**Comment 10:**

In the sentence, “The chemical compositions of both materials were examined using standard spectroscopic techniques to verify compliance with industry-grade requirements,” the authors should indicate which specific spectroscopic techniques were employed, including the brand and model of the equipment used. It should also be clarified whether the materials were produced by the authors or purchased commercially. In either case, complete details regarding material origin and characterization procedures must be provided.

**Response:**

We sincerely thank the reviewer for this valuable suggestion. The manuscript was revised to include additional clarification regarding the origin and initial condition of the investigated materials together with the compositional characterization procedure employed prior to erosion testing. The relevant section now specifies that the materials were commercially procured and that chemical composition verification was performed using optical emission spectroscopy prior to experimental evaluation.

**Comment 11:**

In the phrase, “to the slurry getting old and settling,” the intended meaning is ambiguous. The current wording suggests that the slurry itself is aging, whereas the reviewer assumes that the authors are referring to the equipment aging. This statement should be revised for clarity to accurately reflect the intended subject.

**Response:**

We appreciate the reviewer for identifying this ambiguity. The statement was revised and clarified to accurately describe slurry particle settling and non-uniform particle distribution during prolonged recirculation conditions rather than equipment aging.

**Comment 12:**

The rationale for selecting two slurry concentrations that are very similar, 0.1 wt.% and 0.2 wt.%, should be clearly explained. It is not evident why these specific concentrations were chosen instead of higher values, such as 10 wt.%. The authors should clarify whether higher slurry concentrations can be used with the current setup. If higher concentrations are not feasible, this represents a significant limitation of the equipment, particularly since slurry erosion tests intended to simulate field conditions typically involve much higher particle concentrations. This limitation should be explicitly acknowledged and discussed so that readers can accurately assess the applicability of the setup.

**Response:**

We sincerely thank the reviewer for this important technical observation. Additional clarification regarding the selection of slurry concentrations was incorporated into the revised manuscript. The relatively low slurry concentrations of 0.1 wt.% and 0.2 wt.% were intentionally selected during the preliminary laboratory-scale evaluation stage to maintain controlled particle entrainment conditions while minimizing excessive particle settling, agglomeration, and flow instability within the recirculating slurry system. The revised manuscript also explicitly acknowledges that the present setup has not yet been validated under higher slurry concentration regimes representative of severe industrial slurry environments. Accordingly, this limitation and the need for future evaluation under broader hydrodynamic and high-concentration operating conditions were clearly discussed in the revised manuscript.

**Comment 13:**

All ASTM standards cited in the manuscript must be properly referenced as formal citations. Each standard should be cited within the text (for example, “ASTM E384 [xx]”) and accompanied by complete bibliographic information in the reference list. The authors are advised to verify and correct all ASTM references throughout the manuscript to ensure consistency and compliance with citation standards.

**Response:**

We sincerely appreciate the reviewer’s observation. All ASTM standards cited in the manuscript were carefully verified, properly formatted, and included as formal references within both the manuscript text and reference list to improve consistency and compliance with journal formatting requirements.

**Comment 14:**

The manuscript lacks of hierarchy, please ensure that the sections are numbered and the subsection numbered as well. For example, it should be “3. Experimental Procedure” and “3.1. Test Standards and Protocol”. Similar approach and revision is required throughout the text.

**Response:**

The reviewer’s recommendation has been carefully implemented. The manuscript was thoroughly reorganized with consistent section and subsection numbering throughout the document to improve hierarchy, readability, and structural clarity.

**Comment 15:**

Certain portions of the Results and Discussion section are more appropriately placed within the Methodology section. For example, the following description “Vickers microhardness tests were conducted on brass and cast-iron specimens to evaluate their intrinsic resistance to erosion. Measurements were conducted at three distinct locations on the polished surface of each specimen, and the average results were calculated to assure uniformity and address any local heterogeneities. The test was performed with a load of 1000 g and a dwell time of 30 seconds, in accordance with established microhardness testing standards” is methodological in nature rather than interpretative. The authors should carefully review the Results and Discussion section and relocate similar procedural descriptions to the appropriate part of the manuscript to ensure logical flow and structural consistency.

**Response:**

We sincerely thank the reviewer for this constructive observation. The manuscript was carefully reviewed and reorganized to improve logical flow between methodology and discussion sections. Several procedural and experimental descriptions were relocated from the Results and Discussion section to the appropriate methodology-related subsections to improve structural consistency and readability.

**Comment 16:**

The conclusion section does not adhere to the conventional structure typically required for journal publications. Generally, this section should begin with a brief summary of the study’s objectives and methodology, followed by three to six concise and clearly articulated key conclusions. In its current form, the conclusion is overly extensive, which makes it difficult for readers to identify and understand the main findings of the work.

**Response:**

We sincerely appreciate the reviewer’s valuable recommendation. The conclusion section was substantially revised, condensed, and reorganized in a more conventional scientific format. The revised conclusion now begins with a concise summary of the study objectives and methodology, followed by clearly structured key findings related to apparatus performance, slurry erosion behaviour, wear scar characteristics, and observed ductile and brittle erosion mechanisms under the investigated conditions.
