# Supplementary material for: Development and preliminary experimental evaluation of a venturi-based slurry jet erosion test rig for comparative study of ductile and brittle materials
Source: PLoS One. 2026 Jul 31;21(7):e0355225. doi: 10.1371/journal.pone.0355225 (PMC13426928; doi:10.1371/journal.pone.0355225)
Supplement: Attachment — Submitted filename: Comments -revision.docx [file pone.0355225.s004.docx]

The authors have incorporated most of the suggestions; however, some points still need to be addressed before the manuscript can be accepted.

1. Figure 1 can be presented as a line diagram. The nozzle and stirrer can be illustrated properly. A high-quality image of the “actual slurry erosion setup” should be added, and the key components should be labeled.
2. In the revised manuscript, line 235, the authors ascertained that the developed device was a recirculation type; however, in line 264, they mentioned as follows: “The slurry concentration was controlled by varying the mass flow rate of dry erodent particles introduced through the feeding funnel while maintaining constant water discharge conditions.” On the one hand, the slurry concentration is controlled by the feeding funnel; on the other hand, it has been noted that the particles were allowed to recirculate. There is an ambiguity regarding how the particle concentration is controlled.
3. Please add an optical microscopy or SEM image of brass and cast iron to understand the microstructure.

4. Kindly add SEM and EDS analysis of erodent particles. Surface morphology analysis of erodent is necessary because particle morphology influences the erosion behaviour of materials.

5. Figure 10 Surface roughness of Brass vs. test duration (30 minutes) and 12 Surface roughness of CI vs. test duration (30 minutes): Check the Y axis title.
6. Photographs depicting the erosion scar on the brass and cast-iron samples at different impingement angles will strengthen the manuscript.

7. The erosion scar of a 30° impingement angle looks elliptical in shape, whereas a 90° impingement angle imparts a circular scar. How were the weight loss measurements made? Since the unit mentioned mg/mm^2^, which area was considered? Total area of the sample or the actual eroded area? Explain how the area is calculated and provide the calculations.
8. In the eroded surface morphology, SEM images label the actual erosion mechanisms.

9. In SEM images, the scale bar is not visible properly. Add a scale bar using ImageJ.
10. Figure 14(a) shows deep grooves and craters. Were they material defects or induced by erodent particles? Figure 14(d) shows white patches. What are they? Kindly add EDS analysis and elemental mapping to better understand the eroded surfaces.
