# Supplementary material for: Development and preliminary experimental evaluation of a venturi-based slurry jet erosion test rig for comparative study of ductile and brittle materials
Source: PLoS One. 2026 Jul 31;21(7):e0355225. doi: 10.1371/journal.pone.0355225 (PMC13426928; doi:10.1371/journal.pone.0355225)
Supplement: Attachment — Submitted filename: response letter.docx [file pone.0355225.s005.docx]

Dear Editor and Reviewer,

We sincerely thank the reviewer for the careful evaluation of our manuscript and for the constructive comments and suggestions. We greatly appreciate the time and effort devoted to reviewing our work. The comments have helped us improve the clarity, technical quality, and presentation of the manuscript. All comments have been carefully considered, and the manuscript has been revised accordingly. Our detailed responses are provided below.

**Reviewer -1**

**Comment 1-**“Figure 1 can be presented as a line diagram. The nozzle and stirrer can be illustrated properly. A high-quality image of the”actual slurry erosion setup” should be added, and the key components should be labeled.”

**Response:**

We sincerely thank the reviewer for this valuable suggestion. We respectfully note that Figure 1 presents a classification of commonly reported slurry jet erosion configurations from the literature and has been retained for this purpose. However, to improve the presentation of the developed apparatus, Figure 3 has been substantially revised. A detailed schematic representation of the developed Venturi-based slurry jet erosion rig has been included as Figure 3(a), clearly illustrating the nozzle, particle feeding arrangement, slurry circulation path, specimen holder, and associated components. In addition, a high-quality photograph of the actual laboratory-scale setup has been added as Figure 3(b), with all major components clearly identified and labelled. We believe these revisions significantly improve the clarity and reproducibility of the experimental methodology.

**Comment 2-**“In the revised manuscript, line 235, the authors ascertained that the developed device was a recirculation type; however, in line 264, they mentioned as follows:”The slurry concentration was controlled by varying the mass flow rate of dry erodent particles introduced through the feeding funnel while maintaining constant water discharge conditions.” On the one hand, the slurry concentration is controlled by the feeding funnel; on the other hand, it has been noted that the particles were allowed to recirculate. There is an ambiguity regarding how the particle concentration is controlled.”

**Response:**

We sincerely thank the reviewer for identifying this ambiguity. We agree that clarification of the slurry concentration control procedure is important. Accordingly, Section 2.3.2 has been revised to provide a clearer explanation of the methodology. The revised text now states that the desired slurry concentration was established before each experiment by introducing a predetermined mass of erodent particles into a known volume of water. Once introduced, the particles continuously recirculated within the closed-loop system throughout the test duration. Mechanical stirring was maintained continuously to minimise sedimentation and to promote approximately uniform particle distribution. No additional particles were added during testing. Therefore, the slurry concentration was governed by the initial particle mass-to-water ratio, while the feeding funnel served only as the particle entrainment point into the Venturi stream.

**Comment 3-**“Please add an optical microscopy or SEM image of brass and cast iron to understand the microstructure.”

**Response:**

We sincerely thank the reviewer for this important suggestion. To address this comment, representative pre-erosion SEM microstructures of both brass and cast iron have been included as a new Figure 2. In addition, a dedicated subsection entitled “Initial Microstructural Characteristics” (Section 2.1.1) has been added. The discussion now describes the comparatively homogeneous metallic matrix of brass and the graphite flakes distributed within the predominantly pearlitic-ferrous matrix of grey cast iron. These observations provide a metallurgical basis for understanding the contrasting ductile and brittle erosion mechanisms observed during slurry erosion testing.

**Comment 4-**“Kindly add SEM and EDS analysis of erodent particles. Surface morphology analysis of erodent is necessary because particle morphology influences the erosion behaviour of materials.”

**Response:**

We sincerely thank the reviewer for this insightful comment. We fully agree that erodent morphology plays a significant role in slurry erosion behaviour. Accordingly, SEM micrographs of the river sand particles used in the experiments have been added as Figure 4. The images show irregular angular particles with rough surface features, which are characteristic of naturally occurring abrasive sand particles and are expected to contribute significantly to erosive wear.

With regard to EDS analysis, we agree that compositional characterization would provide useful supplementary information. However, access to EDS facilities was not available during the present investigation. We have therefore transparently acknowledged this limitation in the revised manuscript. Since the primary objective of the present study was to evaluate the performance of the developed slurry erosion rig and investigate erosion behaviour under controlled conditions, emphasis was placed on particle morphology, size, and angularity, which are the dominant physical factors governing mechanical erosion behaviour.

**Comment 5-**“Figure 10 Surface roughness of Brass vs. test duration (30 minutes) and 12 Surface roughness of CI vs. test duration (30 minutes): Check the Y axis title.”

**Response:**

We sincerely thank the reviewer for carefully identifying this error. The Y-axis labels in Figures 10 and 12 have been reviewed and corrected to accurately represent the measured surface roughness parameter. The figures have been updated accordingly.

**Comment 6-**“Photographs depicting the erosion scar on the brass and cast-iron samples at different impingement angles will strengthen the manuscript.”

**Response:**

We sincerely thank the reviewer for this constructive suggestion. To address this comment, a new figure showing representative wear scar morphologies has been added as Figure 15. The figure presents macroscopic images of the erosion scars developed under characteristic ductile and brittle erosion conditions. The brass specimen exhibits an elongated elliptical wear scar consistent with low-angle erosion dominated by micro-cutting and ploughing, whereas the cast iron specimen exhibits a more localized and approximately circular wear scar associated with brittle fracture and crater formation under near-normal impact conditions.

**Comment 7-**“The erosion scar of a 30° impingement angle looks elliptical in shape, whereas a 90° impingement angle imparts a circular scar. How were the weight loss measurements made? Since the unit mentioned mg/mm², which area was considered? Total area of the sample or the actual eroded area? Explain how the area is calculated and provide the calculations.”

**Response:**

We sincerely thank the reviewer for requesting this important methodological clarification. The manuscript has been revised to explicitly state that erosion rates were calculated using the actual projected eroded scar area rather than the total specimen surface area. The calculation methodology has also been added to Section 2.3.2. For elliptical erosion scars formed at lower impingement angles, the projected area was calculated using A = πab, where a and b represent the semi-major and semi-minor axes, respectively. For approximately circular scars formed under near-normal impact conditions, the projected area was calculated using A = πr², where r represents the measured scar radius. The calculated projected area was then used to normalize the measured weight loss and determine erosion rate values expressed in mg/mm².

**Comment 8-**“In the eroded surface morphology, SEM images label the actual erosion mechanisms.”

**Response:**

We sincerely thank the reviewer for this helpful recommendation. The SEM images presented in Figures 16 and 17 have been revised to include clear annotations identifying the dominant erosion mechanisms observed on the surfaces. Features such as groove formation, micro-cutting marks, ploughing, crater formation, crack initiation, crack propagation, brittle fracture regions, and debris accumulation have been labelled directly on the micrographs to improve interpretation and facilitate understanding of the erosion processes.

**Comment 9-**“In SEM images, the scale bar is not visible properly. Add a scale bar using ImageJ.”

**Response:**

We sincerely thank the reviewer for identifying this presentation issue. All SEM micrographs have been revised, and clearly visible scale bars have been incorporated to improve dimensional interpretation. The scale information is now presented more prominently to ensure readability and consistency throughout the manuscript.

**Comment 10-**“Figure 14(a) shows deep grooves and craters. Were they material defects or induced by erodent particles? Figure 14(d) shows white patches. What are they? Kindly add EDS analysis and elemental mapping to better understand the eroded surfaces.”

**Response:**

We sincerely thank the reviewer for this detailed observation. The discussion of SEM morphology has been expanded to clarify the origin of these features. The grooves, craters, fracture regions, and associated surface damage are erosion-induced features generated by repeated abrasive particle impacts and are not pre-existing material defects. Furthermore, the bright or white regions observed in certain SEM micrographs are attributed to compacted erosion debris, localized surface smearing, and topographical contrast effects associated with secondary electron imaging.

We agree that EDS and elemental mapping would provide additional compositional insight regarding adhered debris and localized surface features. However, such facilities were not available during the present investigation. This limitation has now been clearly acknowledged in the revised manuscript. Nevertheless, the SEM observations, erosion trends, impingement-angle dependence, and established erosion mechanisms reported in the literature provide a reliable basis for interpretation of the observed erosion behaviour.

Once again, we sincerely thank the reviewer for the constructive comments and valuable suggestions, which have significantly improved the quality and clarity of the manuscript.

**Responses to Reviewer -2**

**Reviewer Comment:**

*“All revisions have been adequately addressed with detailed and technically sound explanations. The authors' revisions have led to a significant improvement in the manuscript. I recommend publishing the current version.”*

**Author Response:**

We sincerely thank the reviewer for the careful evaluation of the revised manuscript and for the encouraging comments. We are grateful for your positive assessment of our work and for recognizing the efforts made to address the previous review comments. Your recommendation for publication is highly appreciated and serves as valuable encouragement for our research team. We thank you once again for your time, expertise, and constructive feedback throughout the review process.

**Responses to Reviewer -3**

**Reviewer Comment 1:**

*“1. The quality of the figures remains inadequate. All figures appear blurred, even when the original .tif files are downloaded. Although the files meet the specified resolution requirements (dpi), the images are clearly not suitable for publication. The authors should carefully review the PDF generated by the submission platform prior to submission, where it becomes evident that the current figures lack sufficient clarity for publication. In fact, they do not meet the basic and minimum requirements.”*

**Author Response:**

We sincerely thank the reviewer for highlighting this important issue and apologize for the inadequate figure quality in the previous submission. Following your recommendation, all figures were carefully re-examined and regenerated using higher-quality source files in accordance with the journal’s NAAS tool assessment requirements. The revised figures were exported at publication-quality resolution, and all text labels, axes, annotations, scale bars, and image features were individually checked for clarity and readability. In addition, the complete manuscript PDF generated by the submission platform was thoroughly reviewed prior to resubmission to ensure that all figures remained clear and legible in the final compiled version. We believe that the revised figures now meet the visual quality standards required for publication.

**Reviewer Comment 2:**

*“2. The newly introduced Figure 3 raises additional concerns to the whole manuscript. The different regions or components are not clearly identified, and the image includes a corroded tank without adequate explanation. In its current form, Figure 3 is not suitable for publication.”*

**Author Response:**

We sincerely thank the reviewer for this valuable observation. In response, Figure 3 has been completely revised to improve clarity and technical presentation. The updated figure now consists of two complementary parts: (a) a detailed schematic representation of the developed Venturi-based slurry jet erosion apparatus and (b) a photograph of the actual laboratory-scale experimental setup. Major components, including the reservoir tank, centrifugal pump, bypass valve, Venturi section, particle feeding funnel, nozzle, stirrer, and specimen holder, have been clearly identified and labelled.

Furthermore, we have added a specific explanation in Section 2.2.3 regarding the visible corrosion on the external surface of the reservoir tank. The observed corrosion is superficial and resulted from prolonged exposure to water during routine laboratory operation and storage. No internal degradation affecting slurry circulation, particle entrainment, flow regulation, or erosion measurements was observed during experimentation. We believe these revisions have substantially improved the quality, clarity, and transparency of Figure 3.

**Reviewer Comment 3:**

*“3. The initial condition of the materials has not been properly described. Essential information on the microstructural characterization, figures showing the actual microstructure of the materials, and analysis on elements such as grain size, degree of microstructural refinement or coarseness, graphite morphology and distribution, and matrix characteristics, has not been provided.”*

**Author Response:**

We sincerely thank the reviewer for identifying this important deficiency in the previous version of the manuscript. To address this concern, representative pre-erosion SEM microstructures of both brass and cast iron specimens have been added as a new Figure 2. In addition, a dedicated subsection entitled “Initial Microstructural Characteristics” (Section 2.1.1) has been introduced to provide a detailed description of the initial material conditions prior to erosion testing.

The revised discussion now characterizes the comparatively homogeneous metallic matrix of the brass specimen and the graphite flakes distributed within the predominantly pearlitic-ferrous matrix of the grey cast iron specimen. The morphology and distribution of the graphite flakes have been discussed in relation to their role as local stress concentration sites that promote crack initiation and propagation during erosive particle impact. These observations provide a metallurgical basis for understanding the contrasting erosion behaviour observed for the ductile and brittle materials investigated.

Regarding quantitative grain size measurements and detailed microstructural refinement analysis, we respectfully note that the primary objective of the present study was the development and preliminary experimental evaluation of a Venturi-based slurry jet erosion test rig rather than a comprehensive metallurgical characterization study. Nevertheless, the newly added SEM microstructures and accompanying discussion provide an appropriate microstructural baseline for interpreting the erosion mechanisms and material responses observed during testing. We believe that these additions have significantly strengthened the scientific foundation of the manuscript.
