# Supplementary material for: Development and preliminary experimental evaluation of a venturi-based slurry jet erosion test rig for comparative study of ductile and brittle materials
Source: PLoS One. 2026 Jul 31;21(7):e0355225. doi: 10.1371/journal.pone.0355225 (PMC13426928; doi:10.1371/journal.pone.0355225)
Supplement: Attachment — Submitted filename: Response letter R3.docx [file pone.0355225.s006.docx]

**Response to the Reviewer**

**Dear Editor and Reviewer,**

We sincerely thank the reviewer for the careful re-evaluation of our manuscript and for the constructive comments and suggestions. We greatly appreciate the time and expertise devoted to assessing our work. The reviewer’s comments have helped us to further clarify the scope of the investigation, strengthen the presentation of the material and erodent characterisation, and ensure that the conclusions remain appropriately aligned with the experimental evidence.

We have carefully considered both comments and have substantially revised the manuscript accordingly. In particular, we have clarified the apparatus-focused objective of the study; revised the description and interpretation of the pre-erosion SEM observations; added SEM-based morphological characterisation of the river sand erodent; strengthened the description of the post-erosion SEM analysis; and explicitly acknowledged the absence of quantitative metallography and EDS analysis in the scope and limitations of the study. Our detailed responses are provided below.

**Comment 1**

*Initial SEM microstructures of (a) brass and (b) grey cast iron, along with EDS analysis, must be added. The present images (Fig. 2(a) and (b)) do not appear to be micrographs, and the grain boundaries are difficult to discern.*

**Response:**

We sincerely thank the reviewer for this careful observation. We agree that the previous presentation and description of Fig. 2 could have implied a level of quantitative metallographic characterisation that was neither performed nor intended in the present investigation. We have therefore substantially revised the manuscript to clarify the purpose, interpretation, and limitations of these images.

The primary objective of the present study is the development and preliminary experimental evaluation of a Venturi-based slurry jet erosion test rig and the assessment of its capability to distinguish the comparative erosion responses of representative ductile and brittle materials. The study was not designed to establish quantitative correlations between grain structure, phase distribution, local elemental composition, and erosion rate.

The investigated materials were characterised by optical emission spectroscopy (OES) to determine their bulk elemental compositions and by Vickers microhardness measurements to quantify their comparative mechanical response. Pre- and post-erosion SEM observations were employed qualitatively to document representative initial surface features and to support the interpretation of erosion-induced surface morphologies. We fully acknowledge the reviewer’s point that higher-resolution metallographic examination and localised EDS analysis would be necessary for a study seeking quantitative microstructure–erosion or composition–erosion correlations. However, no conclusions concerning grain size, phase fraction, or local elemental composition are drawn from Fig. 2 or elsewhere in the revised manuscript.

In direct response to the reviewer’s concern, Section 2.1.1 has been revised and retitled *“Initial Material Characterisation.”* The text now explicitly states that Fig. 2 provides representative qualitative pre-erosion observations and that detailed phase identification and localised elemental analysis were outside the scope of the investigation. The interpretation of the erosion behaviour has also been carefully revised to rely on the combined evidence from measured bulk composition, hardness, experimental erosion trends, impingement-angle dependence, and characteristic post-erosion surface morphology.

Furthermore, Section 3.6 now explicitly states that SEM was employed for qualitative identification of erosion-induced morphological features rather than quantitative phase or compositional analysis. The manuscript also clarifies that, because EDS and elemental mapping were not performed, no compositional assignment is made for localised bright regions or adhered surface features. Finally, Section 3.7 explicitly acknowledges the absence of quantitative metallographic and EDS analyses as a limitation and restricts the interpretation of the findings to comparative erosion behaviour under the investigated experimental conditions.

We appreciate the reviewer’s comment because it enabled us to remove potential ambiguity concerning the role of Fig. 2 and to establish a clearer distinction between qualitative surface observations and quantitative metallographic characterisation. We respectfully submit that the revised manuscript now accurately represents the available experimental evidence without making unsupported microstructural or composition-based interpretations, while remaining aligned with the stated apparatus-development and preliminary comparative-evaluation objectives of the study.

**Comment 2**

*Kindly add SEM and EDS analysis of erodent particles. Surface morphology analysis of erodent is necessary because particle morphology influences the erosion behaviour of materials.*

**Response:**

We sincerely thank the reviewer for highlighting the importance of erodent particle morphology in slurry erosion behaviour. We agree that particle morphology is an important factor governing particle–surface interaction and erosion mechanisms.

In direct response to this comment, SEM-based morphological characterisation of the river sand erodent has been incorporated into the revised manuscript and is now presented in Fig. 4. Section 2.3.4 has also been retitled *“Erodent Particle Characterisation and SEM Morphology Analysis”* and substantially expanded. The revised section describes both the sieve-based particle-size analysis and the SEM-observed morphology of the erodent. The SEM examination shows that the river sand particles possess irregular and angular geometries, rough surfaces, and non-uniform edges. The relevance of these morphological characteristics to localised stress concentration, cutting, ploughing, and impact-induced surface damage is now explicitly discussed.

We therefore fully agree with, and have addressed, the reviewer’s request concerning SEM-based surface morphology analysis of the erodent.

Regarding EDS analysis, we acknowledge that it would provide additional information on the local elemental composition of the river sand particles and would be essential for an investigation specifically seeking to establish quantitative relationships between erodent mineralogy and erosion behaviour. The present preliminary study, however, was designed to evaluate the operational capability of the developed Venturi-based apparatus and its ability to distinguish comparative erosion responses under controlled variations in particle size, slurry concentration, and impingement angle. It was not designed to investigate mineral-specific erosion mechanisms or to establish correlations between erodent chemistry and material loss.

Accordingly, the erodent was characterised in terms of the physical parameters directly relevant to the experimental programme: sieve-based particle-size distribution, selected particle fractions, and SEM-observed morphology. Introducing compositional data alone, without a corresponding experimental investigation of mineral-specific erosion behaviour, would not provide a quantitative basis for composition–erosion correlations within the stated objectives of the present work.

Nevertheless, we have taken the reviewer’s concern seriously and have revised the manuscript to state this limitation explicitly. Section 3.7 now acknowledges that EDS characterisation of the erodent was not performed and clarifies that such analysis would be necessary for future studies seeking quantitative correlations among erodent mineralogy, material characteristics, and erosion rate. The Conclusions have likewise been revised to ensure that no composition-dependent erosion claims are made.

We are grateful for the reviewer’s suggestion, which has resulted in a meaningful improvement to the manuscript through the addition of SEM-based erodent morphology analysis and clearer definition of the scope and limitations of the erodent characterisation. We respectfully submit that the revised particle-size and SEM morphology characterisation provides the physical information required for interpretation of the present apparatus-focused comparative erosion experiments, while the absence of EDS is now transparently acknowledged and the conclusions are appropriately restricted to the experimental evidence available.

We sincerely thank the reviewer once again for the constructive comments. We believe that the revisions made in response to these observations have substantially improved the scientific clarity, transparency, and scope definition of the manuscript. We respectfully hope that the revised manuscript and the detailed clarifications provided above satisfactorily address the reviewer’s concerns and may now be considered suitable for publication.
