# Supplementary material for: Development and preliminary experimental evaluation of a venturi-based slurry jet erosion test rig for comparative study of ductile and brittle materials
Source: PLoS One. 2026 Jul 31;21(7):e0355225. doi: 10.1371/journal.pone.0355225 (PMC13426928; doi:10.1371/journal.pone.0355225)
Supplement: Peer review history

# Peer review: Decision Letter 0

**DOI:** 10.1371/journal.pone.0355225.r001

**Authors:** Siddhartha Kar

13 May 2026

PONE-D-26-17906

Development and Experimental Validation of a Venturi-Based Slurry Jet Erosion Test Rig for Comparative Study of Ductile and Brittle Materials

PLOS One

Dear Dr. Agarwal,

Thank you for submitting your manuscript to PLOS ONE. After careful consideration, we feel that it has merit but does not fully meet PLOS ONE’s publication criteria as it currently stands. Therefore, we invite you to submit a revised version of the manuscript that addresses the points raised during the review process.

Please submit your revised manuscript by Jun 27 2026 11:59PM. If you will need more time than this to complete your revisions, please reply to this message or contact the journal office at plosone@plos.org. When you're ready to submit your revision, log on to https://www.editorialmanager.com/pone/ and select the 'Submissions Needing Revision' folder to locate your manuscript file.

Please include the following items when submitting your revised manuscript:

- A letter that responds to each point raised by the academic editor and reviewer(s). You should upload this letter as a separate file labeled 'Response to Reviewers'.
- A marked-up copy of your manuscript that highlights changes made to the original version. You should upload this as a separate file labeled 'Revised Manuscript with Track Changes'.
- An unmarked version of your revised paper without tracked changes. You should upload this as a separate file labeled 'Manuscript'.

If you would like to make changes to your financial disclosure, please include your updated statement in your cover letter. Guidelines for resubmitting your figure files are available below the reviewer comments at the end of this letter.

If applicable, we recommend that you deposit your laboratory protocols in protocols.io to enhance the reproducibility of your results. Protocols.io assigns your protocol its own identifier (DOI) so that it can be cited independently in the future. For instructions see: https://journals.plos.org/plosone/s/submission-guidelines#loc-laboratory-protocols. Additionally, PLOS ONE offers an option for publishing peer-reviewed Lab Protocol articles, which describe protocols hosted on protocols.io. Read more information on sharing protocols at https://plos.org/protocols?utm_medium=editorial-email&utm_source=authorletters&utm_campaign=protocols.

As the corresponding author, your ORCID iD is verified in the submission system and will appear in the published article. PLOS supports the use of ORCID, and we encourage all coauthors to register for an ORCID iD and use it as well. Please encourage your coauthors to verify their ORCID iD within the submission system before final acceptance, as unverified ORCID iDs will not appear in the published article. *Only*  the individual author can complete the verification step; PLOS staff *cannot*  verify ORCID iDs on behalf of authors.

We look forward to receiving your revised manuscript.

Kind regards,

Siddhartha Kar

Academic Editor

PLOS One

Journal Requirements:

When submitting your revision, we need you to address these additional requirements.

1. Please ensure that your manuscript meets PLOS ONE's style requirements, including those for file naming. The PLOS ONE style templates can be found at

https://journals.plos.org/plosone/s/file?id=wjVg/PLOSOne_formatting_sample_main_body.pdf and

https://journals.plos.org/plosone/s/file?id=ba62/PLOSOne_formatting_sample_title_authors_affiliations.pdf

2. Thank you for stating the following in the Acknowledgments Section of your manuscript:

“The authors received no specific funding for this work.”

We note that you have provided funding information that is not currently declared in your Funding Statement. However, funding information should not appear in the Acknowledgments section or other areas of your manuscript. We will only publish funding information present in the Funding Statement section of the online submission form.

Please remove any funding-related text from the manuscript and let us know how you would like to update your Funding Statement. Currently, your Funding Statement reads as follows:

“The author(s) received no specific funding for this work.”

Please include your amended statements within your cover letter; we will change the online submission form on your behalf.

3. In the online submission form you indicate that your data is not available for proprietary reasons and have provided a contact point for accessing this data. Please note that your current contact point is a co-author on this manuscript. According to our Data Policy, the contact point must not be an author on the manuscript and must be an institutional contact, ideally not an individual. Please revise your data statement to a non-author institutional point of contact, such as a data access or ethics committee, and send this to us via return email. Please also include contact information for the third party organization, and please include the full citation of where the data can be found.

4. If the reviewer comments include a recommendation to cite specific previously published works, please review and evaluate these publications to determine whether they are relevant and should be cited. There is no requirement to cite these works unless the editor has indicated otherwise.

Additional Editor Comments:

The reviewers have evaluated the manuscript and based on their feedback it cannot be accepted in its present form. However, I am willing to reconsider my decision if all the comments are satisfactorily addressed.

[Note: HTML markup is below. Please do not edit.]

Reviewers' comments:

Reviewer's Responses to Questions

**Comments to the Author**

1. Is the manuscript technically sound, and do the data support the conclusions?

The manuscript must describe a technically sound piece of scientific research with data that supports the conclusions. Experiments must have been conducted rigorously, with appropriate controls, replication, and sample sizes. The conclusions must be drawn appropriately based on the data presented.

Reviewer #1: Yes

Reviewer #2: Yes

Reviewer #3: Partly

**********

2. Has the statistical analysis been performed appropriately and rigorously?

Reviewer #1: I Don't Know

Reviewer #2: N/A

Reviewer #3: Yes

**********

3. Have the authors made all data underlying the findings in their manuscript fully available?

The PLOS Data policy requires authors to make all data underlying the findings described in their manuscript fully available without restriction, with rare exception (please refer to the Data Availability Statement in the manuscript PDF file). The data should be provided as part of the manuscript or its supporting information, or deposited to a public repository. For example, in addition to summary statistics, the data points behind means, medians and variance measures should be available. If there are restrictions on publicly sharing data—e.g. participant privacy or use of data from a third party—those must be specified.

Reviewer #1: Yes

Reviewer #2: Yes

Reviewer #3: Yes

**********

4. Is the manuscript presented in an intelligible fashion and written in standard English?

PLOS ONE does not copyedit accepted manuscripts, so the language in submitted articles must be clear, correct, and unambiguous. Any typographical or grammatical errors should be corrected at revision, so please note any specific errors here.

Reviewer #1: Yes

Reviewer #2: Yes

Reviewer #3: Yes

**********

5. Review Comments to the Author

Please use the space provided to explain your answers to the questions above. You may also include additional comments for the author, including concerns about dual publication, research ethics, or publication ethics. (Please upload your review as an attachment if it exceeds 20,000 characters)

Reviewer #1: The manuscript has certain errors and suggestions which are in need to be incorporated before the acceptance of the manuscript:

1. The manuscript needs a serious revision by a technical writer. The written language is inconsistent. The irrational use of paraphrasing has deteriorated the readability.

2. In Table 1, the erodent size unit has to be given, and the references need to be properly cited.

3. The objective of the research work should be clearly stated. The author has focused on two diverse things. Design and fabrication of the erosion rig and erosion analysis of materials.

4. Kindly add references in the Cast iron as a brittle material section: “Previous studies [References] show that brittle materials tend to wear out the most at high angles because the impact energy hits them straight on, causing them to break apart and chip.”

5. What do you mean by the term “slurry getting old”?

6. Figure 1 can be presented as a line diagram. The nozzle and stirrer can be illustrated properly.

7. Figure 2 has a bypass valve, and one more valve is also there. What are their functions? What is the nozzle diameter? Kindly add it to the test parameter section. The actual setup picture has to be added along with the schematic diagram.

8. After impingement, the erodent particles are collected in the tank, which is connected to the pump. Does this configuration represent a recirculation-type impingement apparatus? If not, how is it ensured that the used particles re-impinge on the sample?

9. What are the dimensions of erosion test samples? What is the sample preparation procedure? How were the samples extracted? What were the initial conditions of the samples, cast or rolled? Kindly add these details.

10. Specify the type of pump used.

11. Please add an optical microscopy or SEM image of brass and cast iron to understand the microstructure.

12. Use the degree (°) symbol instead of 0 as a superscript.

13. Rephrase the following sentence: “The design follows the basic idea of a Venturi meter, where making the space smaller causes the flow to speed up and the pressure to drop at the narrowest point.”

14. How to vary the wt.% of slurry concentration. Particle induction is solely dependent on the pressure drop at the venturi. How is the flow velocity adjusted?

15. Check the spelling of surface roughness tester. “Tallysurf” or “Talysurf”

16. Indentation resistance is primarily controlled by hardness, not resilience. Check the following sentence and provide a proper citation to corroborate the statement: “More resilient materials generally demonstrate superior resistance to surface deformation and particle indentation, particularly under high-velocity impact scenarios.”

17. Specify the type of Cast iron used. How does an iron matrix become brittle? Check the statement or give the citation to support the statement: “CI has sharp carbides and graphite flakes inside a brittle iron matrix, leading to enhanced resistance to indentation.”

18. Kindly add SEM and EDS analysis of erodent particles. Surface morphology analysis of erodent is necessary because particle morphology influences the erosion behaviour of materials.

19. Check the following sentence and add references to strengthen the statement: “This finding matches what is already known, which shows that ductile materials can wear away with low-energy impacts because of processes like bending and movement of the material.” How does the bending and movement of material help the erosion process?

20. In the following sentence, replace “Zenith” with a suitable word: “As illustrated in Fig 7, weight loss initially escalated and attained a zenith at the 10-minute interval.”

21. Figure 8 Surface roughness of Brass vs. test duration (30 minutes) is missing.

22. Rephrase the following sentence to improve the readability: “An analogous experimental methodology was employed for CI, with assessments performed at a 900-impingement angle, consistent with literature suggesting that maximum attrition in brittle materials transpires under normal impact settings [45].”

23. Check the following sentence: “On the other hand, at a 900- impingement angle, as shown in Fig 13 (c) and Fig 13 (d), the surface has uneven dips, bumps, and areas where the material has flowed.” What material has flowed?

24. Check the following sentence: “At a 300 angle of impact, shown in Figs 14 (a) and 14 (b), the main features are shallow grooves and small cuts, showing that some bending of the material happens at first.” Can a particle impingement cause material to bend? That too with 6 m/s velocity with max. 0.2 wt.% concentration? Explain.

25. Add a subheading “Wear scar analysis” and discuss the scar shape and its relationship with respect to the impingement angle. Photographs depicting the erosion scar will strengthen the manuscript.

26. The erosion scar of a 30° impingement angle looks elliptical in shape, whereas a 90° impingement angle imparts a circular scar. How were the weight loss measurements made? Since the unit mentioned mg/mm2, which area was considered? Total area of the sample or the actual eroded area?

27. In Figure 11, at 0.2 wt.% slurry concentration, 510- and 655-micron particle sizes exhibited the same weight loss for 45 to 75° impingement angles; however, at 90° angle, a variation can be seen. Why this disparity? Explain this phenomenon with suitable references.

28. In the eroded surface morphology SEM images, remove the yellow circles. And label the actual erosion mechanisms.

29. The conclusion section should be precise and contain the outcome of the present work.

30. In SEM images, the scale bar is not visible properly. Add a scale bar using ImageJ.

31. Figure 14(a) shows deep grooves and craters. Were they material defects or induced by erodent particles? Figure 14(d) shows white patches. What are they? Kindly add EDS analysis and elemental mapping to better understand the eroded surfaces.

Reviewer #2: The authors developed a Venturi-based slurry jet erosion test setup. They are attempting to experimentally validate this setup on brass (ductile) and cast iron (brittle) metals. The effects of impingement angle, particle size, and slurry concentration are investigated. In addition, the erosion mechanism is interpreted using SEM and microhardness data in the manuscript. However, The manuscript requires the following revisions.

1. The introduction to the manuscript should explain the reasons for developing the Venturi-based setup. Why is a new Venturi-based testing device necessary?

2. The authors used Thapa for slurry jet erosion. However, sources 24 and 25 in the reference list discuss Kathmandu urbanization. Please check this match.

3. I recommend the following articles related to the test setup, erosion, impingement angle, and surface damage:

• https://doi.org/10.1002/pc.27609 :Systematically addresses the impact angle, the difference between the inner and outer surfaces, and solid particle erosion mechanisms. It can strengthen the question "which surface/geometry/impact condition is important and why?" in the introduction.

• https://doi.org/10.3390/polym15020319 :Shows that erosion behavior varies with surface geometry and manufacturing technique. Comparison with the manuscript is possible.

• https://doi.org/10.1002/pc.30102 :This may be useful for demonstrating the relationship between erosion and particle size and material matrix.

• https://doi.org/10.1016/j.ijpvp.2025.105657 :This considers angular impact, velocity, and flow direction together. It contributes to the justification of experimental parameter selection.

4. In the manuscript, tests were conducted at a velocity of 6 m/s, concentrations of 0.1 and 0.2 wt.%, and three particle sizes. Impingement angles were scanned between 15–90°, but there are errors in the table and text representation. This parameter set is insufficient for the validation claim of the test rig. If possible, I suggest you demonstrate that the test rig also operates stably at different velocity regimes, wider concentration ranges, and more challenging hydrodynamic conditions. There doesn't appear to be a rig validation here. It seems like a comparative test series.

5. In the manuscript, "300" and "900" are written instead of "30°" and "90°". Please correct this. Similarly, in Table 5, the angles are given as "15 to 90 (intervals of 150)".

6. The authors mention repeated experiments and averaged observations. However, the number of repetitions, standard deviations, and error bars are not provided. In addition, was the measurement uncertainty calculated? If not, reports of "maximum erosion", "significant anomaly", or "stable operation" should be softened. Additionally, weight loss and surface roughness results are reported as individual trends. However, since the experimental distribution of variation is not shown, it is difficult to compare the mechanical test results with each other.

7. The authors reported 131.2 VHN for Brass and 342.3 VHN for cast iron. The standard deviation of the B values should be given.

8. The authors present a report in the form of high hardness, low erosion, or higher resistance. However, their own data shows that although cast iron is harder, it undergoes erosion with different fracture mechanisms at certain angles. Hardness alone is not a sufficient explanatory factor. The authors should have also discussed toughness, microstructure, graphite morphology, grain size, and crack propagation.

9. In the manuscript, the particles are assumed to be spherical. Therefore, the threshold energy is estimated with the relationship KE = 1/2 m v^2. However, it is immediately stated that River Sand consists of irregular and angular morphology. The energy calculation made by assuming irregular particles as spheres is insufficient for interpreting the threshold values. Also, how were the thresholds of 0.1135 µJ and 1.1572 µJ found in "pilot trials" experimentally verified? These numbers are not convincing without discussing the effect of particle shape.

10. Only sieve analysis was performed in the manuscript. However, the mineralogy, hardness, density, angular sharpness, roundness (shape factor), and surface topography of River Sand were not reported. In slurry erosion, particle geometry and mineral composition are at least as important as size. Therefore, sieve distribution alone seems insufficient for a "comparative erosion" claim. I recommend at least a SEM image, shape analysis, or a simple angularity assessment.

11. The authors report a venturi, pump, bypass valve, and sample holder. However, the nozzle geometry, throat diameter, specimen-nozzle distance, actual velocity distribution, flow regime, particle feed rate calibration, and how slurry homogeneity is ensured in the setup are not specified. In an "apparatus validation" study, I recommend providing the necessary technical data for engineering validation when giving design ideas.

12. Micro-cutting and ploughing were observed for brass, and cracking and fracture for cast iron in the SEM. These comments are consistent with the literature. However, there is no quantitative analysis on the images. I recommend providing cross-validation with pit density, crack length, debris coverage, surface coverage ratio, or profilometry. Also, it was reported that "EDX was not necessary." In that case, the morphology analysis should have been more systematic and measurable. As it stands, the SEM section is not demonstrative; it is descriptive. Please review this section.

13. The manuscript compares test setups using the Levy, Turenne, Hutchings, and Thapa methods. However, the comparison remains qualitative. There are claims such as "simpler," "cheaper," and "more stable." I suggest you make numerical comparisons for these claims, such as operating cost, clogging frequency, particle delivery uniformity, and energy consumption.

14. The manuscript is useful at the laboratory scale. However, for applications such as hydroturbines, pumps, or pipe inner surfaces, testing high-concentration slurry, a wider speed range, different erodent types, and long-term wear behavior is required. The authors have partially acknowledged this as a limitation. However, this narrows the claim of applicability. Therefore, soften the phrase "industrial applicability."

15. There are some problems with English expressions in the manuscript. Phrases such as "You can modify...", "After this research...", "the drop-in erosion rate...", and "tiny cracks and breaking" are not suitable for scientific expression. Additionally, the sentences often contain too much colloquial language or give the impression of being direct software translations. There is a lack of terminological consistency. "Cast iron," "CI," and "brittle alloy" are used interchangeably. Please check and correct this.

Reviewer #3: The authors propose a new slurry jet erosion tester based on the venturi principle. To validate the performance of the proposed device, representative ductile and brittle materials were employed. However, the manuscript does not meet the quality standards required for publication. The following comments and concerns require careful and thorough revision:

1. The authors do not clearly demonstrate how the proposed experimental setup ensures consistent, repeatable, and stable flow conditions, or uniform particle impact. Previous studies have already achieved high reproducibility and consistency using established slurry jet erosion methods. For example, “Slurry Jet Erosion Resistance of VC-Containing Tool Steels: The Role of the Carbide Volume Fraction” (Journal of Materials Engineering and Performance, 2023, 32(24): 11347–11366) reported reliable and repeatable results for both ductile and brittle materials. Therefore, the claimed novelty of the venturi-based test is not evident unless these aspects are explicitly discussed. The authors should clearly explain how this design improves velocity control, consistency, and accuracy compared to existing systems. Additionally, potential issues such as cavitation, impingement angle control, and particle impact focus should be addressed to justify the added value and performance advantages of the proposed setup.

2. The initial state of the materials used is of critical importance. It must be clarified whether the alloys were tested in the as-cast, annealed, heat-treated, cold-rolled, or hot-rolled condition. If heat treatment was applied, the specific parameters should be described. Furthermore, the manuscript lacks a description of the microstructure, which is essential in wear studies. Details such as grain size, microstructural refinement or coarseness, graphite morphology and distribution, and matrix characteristics must be provided. These microstructural and processing details are necessary for reproducibility and for a comprehensive understanding of the wear behavior reported in this study.

3. The manuscript lacks a clear hierarchical structure. The authors should ensure that all sections and subsections are properly numbered to enhance organization and readability. For example, the format should follow a logical sequence such as “3. Experimental Procedure” and “3.1 Test Standards and Protocol.” A similar approach should be applied consistently throughout the manuscript to maintain structural coherence and facilitate navigation.

4. In the sentence, “Among the numerous test rigs produced, including slurry pot, slinger, rotating arm, and jet-type rigs,” specific and authoritative references should be provided for each type of apparatus. Without such citations, the statement appears to rely solely on the authors’ assertions rather than on verifiable scientific sources.

5. All figures in the manuscript are of inadequate quality; they appear blurry even when the original Figures.tif files are downloaded. This significantly hinders the interpretation and critical evaluation of the authors’ discussion. High-resolution versions must be provided to ensure that the figures are suitable for publication and can effectively support the presented work.

6. Regarding Figure 1, the representation of the new equipment is insufficient. When introducing a novel experimental setup, readers and reviewers must be able to clearly observe the complete system, followed by detailed views of key components. In its current form, the figure does not allow comprehension of the main innovation nor identification of essential elements such as particle flow paths, the venturi section, and other critical parts. The figure lacks coherence and technical detail, which compromises reproducibility. A comprehensive image of the full setup should be included, along with well-labeled detailed views of the invention. This figure requires careful revision and should not be treated superficially. Consider to combine or better address this gap with Figure 2 as well.

7. The manuscript text should also be closely aligned with the subfigures in Figure 2. At present, the narrative appears disconnected from the visual material, making it unclear which descriptions correspond to specific parts of the figure. The authors should revise the text to ensure clear linkage between the discussion and the respective subfigures.

8. In Table 1, the column labeled “erodent size” does not specify units, which must be included to ensure clarity and reproducibility. Additionally, several cited references do not appear to correspond to the original or foundational works describing the respective methods. The authors should verify and cite the primary sources in which each technique was initially developed. Moreover, the presence of two similar tables (Table 1 and Table 2) appears redundant. Combining them into a single, comprehensive table would likely improve clarity and avoid unnecessary repetition.

9. The sentence, “As contemporary researchers modified these methodologies, they investigated broader operational parameters,” lacks clarity. The authors should specify who “they” refers to in this context.

10. In the sentence, “The chemical compositions of both materials were examined using standard spectroscopic techniques to verify compliance with industry-grade requirements,” the authors should indicate which specific spectroscopic techniques were employed, including the brand and model of the equipment used. It should also be clarified whether the materials were produced by the authors or purchased commercially. In either case, complete details regarding material origin and characterization procedures must be provided.

11. In the phrase, “to the slurry getting old and settling,” the intended meaning is ambiguous. The current wording suggests that the slurry itself is aging, whereas the reviewer assumes that the authors are referring to the equipment aging. This statement should be revised for clarity to accurately reflect the intended subject.

12. The rationale for selecting two slurry concentrations that are very similar, 0.1 wt.% and 0.2 wt.%, should be clearly explained. It is not evident why these specific concentrations were chosen instead of higher values, such as 10 wt.%. The authors should clarify whether higher slurry concentrations can be used with the current setup. If higher concentrations are not feasible, this represents a significant limitation of the equipment, particularly since slurry erosion tests intended to simulate field conditions typically involve much higher particle concentrations. This limitation should be explicitly acknowledged and discussed so that readers can accurately assess the applicability of the setup.

13. All ASTM standards cited in the manuscript must be properly referenced as formal citations. Each standard should be cited within the text (for example, “ASTM E384 [xx]”) and accompanied by complete bibliographic information in the reference list. The authors are advised to verify and correct all ASTM references throughout the manuscript to ensure consistency and compliance with citation standards.

14. The manuscript lacks of hierarchy, please ensure that the sections are numbered and the subsection numbered as well. For example, it should be “3. Experimental Procedure” and “3.1. Test Standards and Protocol”. Similar approach and revision is required throughout the text.

15. Certain portions of the Results and Discussion section are more appropriately placed within the Methodology section. For example, the following description “Vickers microhardness tests were conducted on brass and cast-iron specimens to evaluate their intrinsic resistance to erosion. Measurements were conducted at three distinct locations on the polished surface of each specimen, and the average results were calculated to assure uniformity and address any local heterogeneities. The test was performed with a load of 1000 g and a dwell time of 30 seconds, in accordance with established microhardness testing standards” is methodological in nature rather than interpretative. The authors should carefully review the Results and Discussion section and relocate similar procedural descriptions to the appropriate part of the manuscript to ensure logical flow and structural consistency.

16. The conclusion section does not adhere to the conventional structure typically required for journal publications. Generally, this section should begin with a brief summary of the study’s objectives and methodology, followed by three to six concise and clearly articulated key conclusions. In its current form, the conclusion is overly extensive, which makes it difficult for readers to identify and understand the main findings of the work.

**********

6. PLOS authors have the option to publish the peer review history of their article (what does this mean?). If published, this will include your full peer review and any attached files.

If you choose “no”, your identity will remain anonymous but your review may still be made public.

**Do you want your identity to be public for this peer review?**  For information about this choice, including consent withdrawal, please see our Privacy Policy.

Reviewer #1: No

Reviewer #2: **Yes:** Harun Sepetcioglu

Reviewer #3: No

**********

[NOTE: If reviewer comments were submitted as an attachment file, they will be attached to this email and accessible via the submission site. Please log into your account, locate the manuscript record, and check for the action link "View Attachments". If this link does not appear, there are no attachment files.]

To ensure your figures meet our technical requirements, please review our figure guidelines: https://journals.plos.org/plosone/s/figures

You may also use PLOS’s free figure tool, NAAS, to help you prepare publication quality figures: https://journals.plos.org/plosone/s/figures#loc-tools-for-figure-preparation.

NAAS will assess whether your figures meet our technical requirements by comparing each figure against our figure specifications.

Attachment Submitted filename: Comments.docx

# Author comment: Author response to Decision Letter 1

**DOI:** 10.1371/journal.pone.0355225.r002

24 May 2026

We sincerely thank the Editor and the Reviewers for their careful evaluation of our manuscript and for the valuable technical comments and constructive suggestions provided. We greatly appreciate the time and effort invested in reviewing our work.

In response to the reviewer and editor recommendations, the manuscript has been thoroughly revised throughout to improve scientific clarity, technical consistency, experimental description, figure quality, and overall presentation. Major revisions include comprehensive language refinement, restructuring of several sections, clarification of the objectives and scope of the study, addition of detailed apparatus specifications, inclusion of high-resolution setup and SEM images, improved wear scar and erosion mechanism discussion, correction of figure formatting issues, verification and correction of references and ASTM citations, and moderation of terminology related to validation and industrial applicability.

In addition, the Data Availability Statement has been revised in accordance with PLOS data policy requirements, and the institutional contact information has been updated accordingly.

All reviewer comments have been addressed carefully on a point-by-point basis, and detailed responses have been provided in the revised response-to-reviewers document.

We sincerely thank the Editor and Reviewers once again for their valuable feedback, which significantly helped improve the quality and clarity of the manuscript.

Attachment Submitted filename: Response to Reviewers.docx

# Peer review: Decision Letter 1

**DOI:** 10.1371/journal.pone.0355225.r003

**Authors:** Siddhartha Kar

11 Jun 2026

PONE-D-26-17906R1

Development and Preliminary Experimental Evaluation of a Venturi-Based Slurry Jet Erosion Test Rig for Comparative Study of Ductile and Brittle Materials

PLOS One

Dear Dr. Agarwal,

Thank you for submitting your manuscript to PLOS ONE. After careful consideration, we feel that it has merit but does not fully meet PLOS ONE’s publication criteria as it currently stands. Therefore, we invite you to submit a revised version of the manuscript that addresses the points raised during the review process.

Please submit your revised manuscript by Jul 26 2026 11:59PM. If you will need more time than this to complete your revisions, please reply to this message or contact the journal office at plosone@plos.org. When you're ready to submit your revision, log on to https://www.editorialmanager.com/pone/ and select the 'Submissions Needing Revision' folder to locate your manuscript file.

Please include the following items when submitting your revised manuscript:

- A letter that responds to each point raised by the academic editor and reviewer(s). You should upload this letter as a separate file labeled 'Response to Reviewers'.
- A marked-up copy of your manuscript that highlights changes made to the original version. You should upload this as a separate file labeled 'Revised Manuscript with Track Changes'.
- An unmarked version of your revised paper without tracked changes. You should upload this as a separate file labeled 'Manuscript'.

If you would like to make changes to your financial disclosure, please include your updated statement in your cover letter. Guidelines for resubmitting your figure files are available below the reviewer comments at the end of this letter.

If applicable, we recommend that you deposit your laboratory protocols in protocols.io to enhance the reproducibility of your results. Protocols.io assigns your protocol its own identifier (DOI) so that it can be cited independently in the future. For instructions see: https://journals.plos.org/plosone/s/submission-guidelines#loc-laboratory-protocols. Additionally, PLOS ONE offers an option for publishing peer-reviewed Lab Protocol articles, which describe protocols hosted on protocols.io. Read more information on sharing protocols at https://plos.org/protocols?utm_medium=editorial-email&utm_source=authorletters&utm_campaign=protocols.

As the corresponding author, your ORCID iD is verified in the submission system and will appear in the published article. PLOS supports the use of ORCID, and we encourage all coauthors to register for an ORCID iD and use it as well. Please encourage your coauthors to verify their ORCID iD within the submission system before final acceptance, as unverified ORCID iDs will not appear in the published article. *Only*  the individual author can complete the verification step; PLOS staff *cannot*  verify ORCID iDs on behalf of authors.

We look forward to receiving your revised manuscript.

Kind regards,

Siddhartha Kar

Academic Editor

PLOS One

Journal Requirements:

If the reviewer comments include a recommendation to cite specific previously published works, please review and evaluate these publications to determine whether they are relevant and should be cited. There is no requirement to cite these works unless the editor has indicated otherwise.

Additional Editor Comments:

Kindly address the reviewers' comments.

[Note: HTML markup is below. Please do not edit.]

Reviewers' comments:

Reviewer's Responses to Questions

**Comments to the Author**

1. If the authors have adequately addressed your comments raised in a previous round of review and you feel that this manuscript is now acceptable for publication, you may indicate that here to bypass the “Comments to the Author” section, enter your conflict of interest statement in the “Confidential to Editor” section, and submit your "Accept" recommendation.

Reviewer #1: (No Response)

Reviewer #2: All comments have been addressed

Reviewer #3: (No Response)

**********

2. Is the manuscript technically sound, and do the data support the conclusions?

The manuscript must describe a technically sound piece of scientific research with data that supports the conclusions. Experiments must have been conducted rigorously, with appropriate controls, replication, and sample sizes. The conclusions must be drawn appropriately based on the data presented.

Reviewer #1: Yes

Reviewer #2: Yes

Reviewer #3: Partly

**********

3. Has the statistical analysis been performed appropriately and rigorously?

Reviewer #1: I Don't Know

Reviewer #2: N/A

Reviewer #3: N/A

**********

4. Have the authors made all data underlying the findings in their manuscript fully available?

The PLOS Data policy requires authors to make all data underlying the findings described in their manuscript fully available without restriction, with rare exception (please refer to the Data Availability Statement in the manuscript PDF file). The data should be provided as part of the manuscript or its supporting information, or deposited to a public repository. For example, in addition to summary statistics, the data points behind means, medians and variance measures should be available. If there are restrictions on publicly sharing data—e.g. participant privacy or use of data from a third party—those must be specified.

Reviewer #1: Yes

Reviewer #2: Yes

Reviewer #3: Yes

**********

5. Is the manuscript presented in an intelligible fashion and written in standard English?

PLOS ONE does not copyedit accepted manuscripts, so the language in submitted articles must be clear, correct, and unambiguous. Any typographical or grammatical errors should be corrected at revision, so please note any specific errors here.

Reviewer #1: Yes

Reviewer #2: Yes

Reviewer #3: Yes

**********

6. Review Comments to the Author

Please use the space provided to explain your answers to the questions above. You may also include additional comments for the author, including concerns about dual publication, research ethics, or publication ethics. (Please upload your review as an attachment if it exceeds 20,000 characters)

Reviewer #1: The authors have incorporated most of the suggestions; however, some points still need to be addressed before the manuscript can be accepted. Comments file is attached.

Reviewer #2: All revisions have been adequately addressed with detailed and technically sound explanations. The authors' revisions have led to a significant improvement in the manuscript. I recommend publishing the current version.

Reviewer #3: The authors have not adequately addressed the reviewer’s concerns or provided sufficient clarification. Only minor modifications appear to have been made in the revised manuscript in response to the comments. The reviewer will not reiterate all previous remarks but will highlight key issues that remain unresolved and that limit the suitability of this manuscript for publication:

1. The quality of the figures remains inadequate. All figures appear blurred, even when the original .tif files are downloaded. Although the files meet the specified resolution requirements (dpi), the images are clearly not suitable for publication. The authors should carefully review the PDF generated by the submission platform prior to submission, where it becomes evident that the current figures lack sufficient clarity for publication. In fact, they do not meet the basic and minimum requirements.

2. The newly introduced Figure 3 raises additional concerns to the whole manuscript. The different regions or components are not clearly identified, and the image includes a corroded tank without adequate explanation. In its current form, Figure 3 is not suitable for publication.

3. The initial condition of the materials has not been properly described. Essential information on the microstructural characterization, figures showing the actual microstructure of the materials, and analysis on elements such as grain size, degree of microstructural refinement or coarseness, graphite morphology and distribution, and matrix characteristics, has not been provided.

**********

7. PLOS authors have the option to publish the peer review history of their article (what does this mean?). If published, this will include your full peer review and any attached files.

If you choose “no”, your identity will remain anonymous but your review may still be made public.

**Do you want your identity to be public for this peer review?**  For information about this choice, including consent withdrawal, please see our Privacy Policy.

Reviewer #1: No

Reviewer #2: **Yes:** Harun SEPETCIOGLU

Reviewer #3: No

**********

[NOTE: If reviewer comments were submitted as an attachment file, they will be attached to this email and accessible via the submission site. Please log into your account, locate the manuscript record, and check for the action link "View Attachments". If this link does not appear, there are no attachment files.]

To ensure your figures meet our technical requirements, please review our figure guidelines: https://journals.plos.org/plosone/s/figures

You may also use PLOS’s free figure tool, NAAS, to help you prepare publication quality figures: https://journals.plos.org/plosone/s/figures#loc-tools-for-figure-preparation.

NAAS will assess whether your figures meet our technical requirements by comparing each figure against our figure specifications.

Attachment Submitted filename: Comments -revision.docx

# Author comment: Author response to Decision Letter 2

**DOI:** 10.1371/journal.pone.0355225.r004

15 Jun 2026

We sincerely thank the Editor and Reviewers for their careful evaluation and constructive comments. All reviewer comments have been addressed in the revised manuscript and detailed responses have been provided in the accompanying Response to Reviewers document.

Major revisions include: (i) regeneration and improvement of all figures to enhance publication quality and readability; (ii) revision of the slurry erosion test rig description with improved schematic and labelled experimental setup; (iii) addition of pre-erosion SEM microstructures of brass and cast iron together with a dedicated microstructural characterization section; (iv) inclusion of SEM morphology of erodent particles; (v) addition of representative wear scar images and clarification of erosion area calculations; (vi) revision and annotation of SEM erosion micrographs with improved scale bars and erosion mechanism identification; and (vii) clarification of SEM-observed surface features, including the interpretation of bright regions (white patches), together with explicit discussion of study limitations regarding EDS analysis.

We believe that the revisions have substantially improved the clarity, technical quality, and scientific presentation of the manuscript. We respectfully submit the revised manuscript for your consideration and sincerely appreciate the time and effort invested by the Editor and Reviewers throughout the review process.

Attachment Submitted filename: response letter.docx

# Peer review: Decision Letter 2

**DOI:** 10.1371/journal.pone.0355225.r005

**Authors:** Siddhartha Kar

1 Jul 2026

PONE-D-26-17906R2

Development and Preliminary Experimental Evaluation of a Venturi-Based Slurry Jet Erosion Test Rig for Comparative Study of Ductile and Brittle Materials

PLOS One

Dear Dr. Agarwal,

Thank you for submitting your manuscript to PLOS ONE. After careful consideration, we feel that it has merit but does not fully meet PLOS ONE’s publication criteria as it currently stands. Therefore, we invite you to submit a revised version of the manuscript that addresses the points raised during the review process.

Please submit your revised manuscript by  Aug 15 2026 11:59PM. If you will need more time than this to complete your revisions, please reply to this message or contact the journal office at plosone@plos.org. When you're ready to submit your revision, log on to https://www.editorialmanager.com/pone/ and select the 'Submissions Needing Revision' folder to locate your manuscript file.

Please include the following items when submitting your revised manuscript:

- A letter that responds to each point raised by the academic editor and reviewer(s). You should upload this letter as a separate file labeled 'Response to Reviewers'.
- A marked-up copy of your manuscript that highlights changes made to the original version. You should upload this as a separate file labeled 'Revised Manuscript with Track Changes'.
- An unmarked version of your revised paper without tracked changes. You should upload this as a separate file labeled 'Manuscript'.

If you would like to make changes to your financial disclosure, please include your updated statement in your cover letter. Guidelines for resubmitting your figure files are available below the reviewer comments at the end of this letter.

If applicable, we recommend that you deposit your laboratory protocols in protocols.io to enhance the reproducibility of your results. Protocols.io assigns your protocol its own identifier (DOI) so that it can be cited independently in the future. For instructions see: https://journals.plos.org/plosone/s/submission-guidelines#loc-laboratory-protocols. Additionally, PLOS ONE offers an option for publishing peer-reviewed Lab Protocol articles, which describe protocols hosted on protocols.io. Read more information on sharing protocols at https://plos.org/protocols?utm_medium=editorial-email&utm_source=authorletters&utm_campaign=protocols.

As the corresponding author, your ORCID iD is verified in the submission system and will appear in the published article. PLOS supports the use of ORCID, and we encourage all coauthors to register for an ORCID iD and use it as well. Please encourage your coauthors to verify their ORCID iD within the submission system before final acceptance, as unverified ORCID iDs will not appear in the published article. *Only*  the individual author can complete the verification step; PLOS staff *cannot*  verify ORCID iDs on behalf of authors.

We look forward to receiving your revised manuscript.

Kind regards,

Siddhartha Kar

Academic Editor

PLOS One

Journal Requirements:

If the reviewer comments include a recommendation to cite specific previously published works, please review and evaluate these publications to determine whether they are relevant and should be cited. There is no requirement to cite these works unless the editor has indicated otherwise.

Additional Editor Comments:

Kindly address the reviewers' comments.

[Note: HTML markup is below. Please do not edit.]

Reviewers' comments:

Reviewer's Responses to Questions

**Comments to the Author**

1. If the authors have adequately addressed your comments raised in a previous round of review and you feel that this manuscript is now acceptable for publication, you may indicate that here to bypass the “Comments to the Author” section, enter your conflict of interest statement in the “Confidential to Editor” section, and submit your "Accept" recommendation.

Reviewer #1: (No Response)

Reviewer #2: All comments have been addressed

**********

2. Is the manuscript technically sound, and do the data support the conclusions?

The manuscript must describe a technically sound piece of scientific research with data that supports the conclusions. Experiments must have been conducted rigorously, with appropriate controls, replication, and sample sizes. The conclusions must be drawn appropriately based on the data presented.

Reviewer #1: Yes

Reviewer #2: Yes

**********

3. Has the statistical analysis been performed appropriately and rigorously?

Reviewer #1: N/A

Reviewer #2: Yes

**********

4. Have the authors made all data underlying the findings in their manuscript fully available?

The PLOS Data policy requires authors to make all data underlying the findings described in their manuscript fully available without restriction, with rare exception (please refer to the Data Availability Statement in the manuscript PDF file). The data should be provided as part of the manuscript or its supporting information, or deposited to a public repository. For example, in addition to summary statistics, the data points behind means, medians and variance measures should be available. If there are restrictions on publicly sharing data—e.g. participant privacy or use of data from a third party—those must be specified.

Reviewer #1: Yes

Reviewer #2: Yes

**********

5. Is the manuscript presented in an intelligible fashion and written in standard English?

PLOS ONE does not copyedit accepted manuscripts, so the language in submitted articles must be clear, correct, and unambiguous. Any typographical or grammatical errors should be corrected at revision, so please note any specific errors here.

Reviewer #1: Yes

Reviewer #2: Yes

**********

6. Review Comments to the Author

Please use the space provided to explain your answers to the questions above. You may also include additional comments for the author, including concerns about dual publication, research ethics, or publication ethics. (Please upload your review as an attachment if it exceeds 20,000 characters)

Reviewer #1: The authors need to incorporate few things before the manuscript can be accepted.

1. Initial SEM microstructures of (a) brass and (b) grey cast iron, along with EDS analysis, must be added. The present images (Fig. 2(a) and (b)) do not appear to be micrographs, and the grain boundaries are difficult to discern.

2. Kindly add SEM and EDS analysis of erodent particles. Surface morphology analysis of erodent is necessary because particle morphology influences the erosion behaviour of materials.

Reviewer #2: Dear Editor,

The authors have successfully addressed the concerns raised. Therefore, I recommend that the manuscript be published.

**********

7. PLOS authors have the option to publish the peer review history of their article (what does this mean?). If published, this will include your full peer review and any attached files.

If you choose “no”, your identity will remain anonymous but your review may still be made public.

**Do you want your identity to be public for this peer review?**  For information about this choice, including consent withdrawal, please see our Privacy Policy.

Reviewer #1: No

Reviewer #2: No

**********

[NOTE: If reviewer comments were submitted as an attachment file, they will be attached to this email and accessible via the submission site. Please log into your account, locate the manuscript record, and check for the action link "View Attachments". If this link does not appear, there are no attachment files.]

To ensure your figures meet our technical requirements, please review our figure guidelines: https://journals.plos.org/plosone/s/figures

You may also use PLOS’s free figure tool, NAAS, to help you prepare publication quality figures: https://journals.plos.org/plosone/s/figures#loc-tools-for-figure-preparation.

NAAS will assess whether your figures meet our technical requirements by comparing each figure against our figure specifications.

# Author comment: Author response to Decision Letter 3

**DOI:** 10.1371/journal.pone.0355225.r006

7 Jul 2026

Dear Editor and Reviewer,

We sincerely thank the reviewer for the careful re-evaluation of our manuscript and for the constructive comments and suggestions. We greatly appreciate the time and expertise devoted to assessing our work. The reviewer’s comments have helped us to further clarify the scope of the investigation, strengthen the presentation of the material and erodent characterisation, and ensure that the conclusions remain appropriately aligned with the experimental evidence.

We have carefully considered both comments and have substantially revised the manuscript accordingly. In particular, we have clarified the apparatus-focused objective of the study; revised the description and interpretation of the pre-erosion SEM observations; added SEM-based morphological characterisation of the river sand erodent; strengthened the description of the post-erosion SEM analysis; and explicitly acknowledged the absence of quantitative metallography and EDS analysis in the scope and limitations of the study. Our detailed responses are provided below.

Comment 1

Initial SEM microstructures of (a) brass and (b) grey cast iron, along with EDS analysis, must be added. The present images (Fig. 2(a) and (b)) do not appear to be micrographs, and the grain boundaries are difficult to discern.

Response:

We sincerely thank the reviewer for this careful observation. We agree that the previous presentation and description of Fig. 2 could have implied a level of quantitative metallographic characterisation that was neither performed nor intended in the present investigation. We have therefore substantially revised the manuscript to clarify the purpose, interpretation, and limitations of these images.

The primary objective of the present study is the development and preliminary experimental evaluation of a Venturi-based slurry jet erosion test rig and the assessment of its capability to distinguish the comparative erosion responses of representative ductile and brittle materials. The study was not designed to establish quantitative correlations between grain structure, phase distribution, local elemental composition, and erosion rate.

The investigated materials were characterised by optical emission spectroscopy (OES) to determine their bulk elemental compositions and by Vickers microhardness measurements to quantify their comparative mechanical response. Pre- and post-erosion SEM observations were employed qualitatively to document representative initial surface features and to support the interpretation of erosion-induced surface morphologies. We fully acknowledge the reviewer’s point that higher-resolution metallographic examination and localised EDS analysis would be necessary for a study seeking quantitative microstructure–erosion or composition–erosion correlations. However, no conclusions concerning grain size, phase fraction, or local elemental composition are drawn from Fig. 2 or elsewhere in the revised manuscript.

In direct response to the reviewer’s concern, Section 2.1.1 has been revised and retitled “Initial Material Characterisation.” The text now explicitly states that Fig. 2 provides representative qualitative pre-erosion observations and that detailed phase identification and localised elemental analysis were outside the scope of the investigation. The interpretation of the erosion behaviour has also been carefully revised to rely on the combined evidence from measured bulk composition, hardness, experimental erosion trends, impingement-angle dependence, and characteristic post-erosion surface morphology.

Furthermore, Section 3.6 now explicitly states that SEM was employed for qualitative identification of erosion-induced morphological features rather than quantitative phase or compositional analysis. The manuscript also clarifies that, because EDS and elemental mapping were not performed, no compositional assignment is made for localised bright regions or adhered surface features. Finally, Section 3.7 explicitly acknowledges the absence of quantitative metallographic and EDS analyses as a limitation and restricts the interpretation of the findings to comparative erosion behaviour under the investigated experimental conditions.

We appreciate the reviewer’s comment because it enabled us to remove potential ambiguity concerning the role of Fig. 2 and to establish a clearer distinction between qualitative surface observations and quantitative metallographic characterisation. We respectfully submit that the revised manuscript now accurately represents the available experimental evidence without making unsupported microstructural or composition-based interpretations, while remaining aligned with the stated apparatus-development and preliminary comparative-evaluation objectives of the study.

Comment 2

Kindly add SEM and EDS analysis of erodent particles. Surface morphology analysis of erodent is necessary because particle morphology influences the erosion behaviour of materials.

Response:

We sincerely thank the reviewer for highlighting the importance of erodent particle morphology in slurry erosion behaviour. We agree that particle morphology is an important factor governing particle–surface interaction and erosion mechanisms.

In direct response to this comment, SEM-based morphological characterisation of the river sand erodent has been incorporated into the revised manuscript and is now presented in Fig. 4. Section 2.3.4 has also been retitled “Erodent Particle Characterisation and SEM Morphology Analysis” and substantially expanded. The revised section describes both the sieve-based particle-size analysis and the SEM-observed morphology of the erodent. The SEM examination shows that the river sand particles possess irregular and angular geometries, rough surfaces, and non-uniform edges. The relevance of these morphological characteristics to localised stress concentration, cutting, ploughing, and impact-induced surface damage is now explicitly discussed.

We therefore fully agree with, and have addressed, the reviewer’s request concerning SEM-based surface morphology analysis of the erodent.

Regarding EDS analysis, we acknowledge that it would provide additional information on the local elemental composition of the river sand particles and would be essential for an investigation specifically seeking to establish quantitative relationships between erodent mineralogy and erosion behaviour. The present preliminary study, however, was designed to evaluate the operational capability of the developed Venturi-based apparatus and its ability to distinguish comparative erosion responses under controlled variations in particle size, slurry concentration, and impingement angle. It was not designed to investigate mineral-specific erosion mechanisms or to establish correlations between erodent chemistry and material loss.

Accordingly, the erodent was characterised in terms of the physical parameters directly relevant to the experimental programme: sieve-based particle-size distribution, selected particle fractions, and SEM-observed morphology. Introducing compositional data alone, without a corresponding experimental investigation of mineral-specific erosion behaviour, would not provide a quantitative basis for composition–erosion correlations within the stated objectives of the present work.

Nevertheless, we have taken the reviewer’s concern seriously and have revised the manuscript to state this limitation explicitly. Section 3.7 now acknowledges that EDS characterisation of the erodent was not performed and clarifies that such analysis would be necessary for future studies seeking quantitative correlations among erodent mineralogy, material characteristics, and erosion rate. The Conclusions have likewise been revised to ensure that no composition-dependent erosion claims are made.

We are grateful for the reviewer’s suggestion, which has resulted in a meaningful improvement to the manuscript through the addition of SEM-based erodent morphology analysis and clearer definition of the scope and limitations of the erodent characterisation. We respectfully submit that the revised particle-size and SEM morphology characterisation provides the physical information required for interpretation of the present apparatus-focused comparative erosion experiments, while the absence of EDS is now transparently acknowledged and the conclusions are appropriately restricted to the experimental evidence available.

We sincerely thank the reviewer once again for the constructive comments. We believe that the revisions made in response to these observations have substantially improved the scientific clarity, transparency, and scope definition of the manuscript. We respectfully hope that the revised manuscript and the detailed clarifications provided above satisfactorily address the reviewer’s concerns and may now be considered suitable for publication.

Attachment Submitted filename: Response letter R3.docx

# Peer review: Decision Letter 3

**DOI:** 10.1371/journal.pone.0355225.r007

**Authors:** Siddhartha Kar

19 Jul 2026

Development and Preliminary Experimental Evaluation of a Venturi-Based Slurry Jet Erosion Test Rig for Comparative Study of Ductile and Brittle Materials

PONE-D-26-17906R3

Dear Dr. Agarwal,

We’re pleased to inform you that your manuscript has been judged scientifically suitable for publication and will be formally accepted for publication once it meets all outstanding technical requirements.

Within one week, you’ll receive an e-mail detailing the required amendments. When these have been addressed, you’ll receive a formal acceptance letter and your manuscript will be scheduled for publication.

An invoice will be generated when your article is formally accepted. Please note, if your institution has a publishing partnership with PLOS and your article meets the relevant criteria, all or part of your publication costs will be covered. Please make sure your user information is up-to-date by logging into Editorial Manager at Editorial Manager® and clicking the ‘Update My Information' link at the top of the page. For questions related to billing, please contact billing support.

If your institution or institutions have a press office, please notify them about your upcoming paper to help maximize its impact. If they’ll be preparing press materials, please inform our press team as soon as possible -- no later than 48 hours after receiving the formal acceptance. Your manuscript will remain under strict press embargo until 2 pm Eastern Time on the date of publication. For more information, please contact onepress@plos.org.

Kind regards,

Siddhartha Kar

Academic Editor

PLOS One

Additional Editor Comments (optional):

The reviewers are satisfied with the incorporated modifications. Thus, I am happy to accept it in its present form.

Reviewers' comments:

Reviewer's Responses to Questions

**Comments to the Author**

1. If the authors have adequately addressed your comments raised in a previous round of review and you feel that this manuscript is now acceptable for publication, you may indicate that here to bypass the “Comments to the Author” section, enter your conflict of interest statement in the “Confidential to Editor” section, and submit your "Accept" recommendation.

Reviewer #1: (No Response)

Reviewer #2: All comments have been addressed

**********

2. Is the manuscript technically sound, and do the data support the conclusions?

The manuscript must describe a technically sound piece of scientific research with data that supports the conclusions. Experiments must have been conducted rigorously, with appropriate controls, replication, and sample sizes. The conclusions must be drawn appropriately based on the data presented.

Reviewer #1: Yes

Reviewer #2: Yes

**********

3. Has the statistical analysis been performed appropriately and rigorously?

Reviewer #1: I Don't Know

Reviewer #2: N/A

**********

4. Have the authors made all data underlying the findings in their manuscript fully available?

The PLOS Data policy requires authors to make all data underlying the findings described in their manuscript fully available without restriction, with rare exception (please refer to the Data Availability Statement in the manuscript PDF file). The data should be provided as part of the manuscript or its supporting information, or deposited to a public repository. For example, in addition to summary statistics, the data points behind means, medians and variance measures should be available. If there are restrictions on publicly sharing data—e.g. participant privacy or use of data from a third party—those must be specified.

Reviewer #1: Yes

Reviewer #2: Yes

**********

5. Is the manuscript presented in an intelligible fashion and written in standard English?

PLOS ONE does not copyedit accepted manuscripts, so the language in submitted articles must be clear, correct, and unambiguous. Any typographical or grammatical errors should be corrected at revision, so please note any specific errors here.

Reviewer #1: Yes

Reviewer #2: Yes

**********

6. Review Comments to the Author

Please use the space provided to explain your answers to the questions above. You may also include additional comments for the author, including concerns about dual publication, research ethics, or publication ethics. (Please upload your review as an attachment if it exceeds 20,000 characters)

Reviewer #1: The authors have responded to the review comments with a textual explanation rather than incorporating the suggested revisions into the manuscript. This is not an ideal approach.

Reviewer #2: The authors have carefully considered the peer review comments. They provided clear, well-reasoned, and scientifically sound corrections. These improvements have significantly enhanced the quality and clarity of the manuscript. The manuscript is suitable for publication in its current form.

**********

7. PLOS authors have the option to publish the peer review history of their article (what does this mean?). If published, this will include your full peer review and any attached files.

If you choose “no”, your identity will remain anonymous but your review may still be made public.

**Do you want your identity to be public for this peer review?**  For information about this choice, including consent withdrawal, please see our Privacy Policy.

Reviewer #1: No

Reviewer #2: No

**********

# Editor report: Acceptance letter

**DOI:** 10.1371/journal.pone.0355225.r008

**Authors:** Siddhartha Kar

PONE-D-26-17906R3

PLOS One

Dear Dr. Agarwal,

I'm pleased to inform you that your manuscript has been deemed suitable for publication in PLOS One. Congratulations! Your manuscript is now being handed over to our production team.

At this stage, our production department will prepare your paper for publication. This includes ensuring the following:

* All references, tables, and figures are properly cited

* All relevant supporting information is included in the manuscript submission,

* There are no issues that prevent the paper from being properly typeset

You will receive further instructions from the production team, including instructions on how to review your proof when it is ready. Please keep in mind that we are working through a large volume of accepted articles, so please give us a few days to review your paper and let you know the next and final steps.

Lastly, if your institution or institutions have a press office, please let them know about your upcoming paper now to help maximize its impact. If they'll be preparing press materials, please inform our press team within the next 48 hours. Your manuscript will remain under strict press embargo until 2 pm Eastern Time on the date of publication. For more information, please contact onepress@plos.org.

You will receive an invoice from PLOS for your publication fee after your manuscript has reached the completed accept phase. If you receive an email requesting payment before acceptance or for any other service, this may be a phishing scheme. Learn how to identify phishing emails and protect your accounts at https://explore.plos.org/phishing.

If we can help with anything else, please email us at customercare@plos.org.

Thank you for submitting your work to PLOS ONE and supporting open access.

Kind regards,

PLOS ONE Editorial Office Staff

on behalf of

Dr. Siddhartha Kar

Academic Editor

PLOS One
